# Structural and functional impact of cystic fibrosis on endocrine pancreatic innervation

**DOI:** 10.1042/CS20260182

**Published:** 2026-09-29

**Authors:** Xingshen Sun, Yaling Yi, Shanming Hu, Gulnara Novbatova, Aliye Uc, Katie Larson Ode, John F. Engelhardt, Andrew W. Norris

**Affiliations:** 1 Pulmonary, Allergy, and Critical Care Medicine, Department of Medicine, University of Alabama at Birmingham, Birmingham, AL 35294, U.S.A.; 2 Stead Family Department of Pediatrics, University of Iowa, Iowa City, IA 52242, U.S.A.; 3 Fraternal Order of Eagle Diabetes Research Center, University of Iowa, Iowa City, IA 52242, U.S.A.; 4 Department of Radiation Oncology, University of Iowa, Iowa City, IA 52242, U.S.A.

**Keywords:** autonomic nervous system, glucagon, neurodegeneration, pancreatic beta cell, pancreatic islets, pancreatic polypeptide

## Abstract

In health, the autonomic nervous system modifies secretion of the islet hormones insulin, glucagon, and pancreatic polypeptide (PP). Uniquely, PP secretion is nearly fully dependent upon parasympathetic innervation. In people with cystic fibrosis (CF), physiological secretion of all three hormones is impaired, but the role of the autonomic nervous system in this dysfunction is unknown. We thus examined PP secretion and pancreatic neuronal immunostaining in humans and ferrets with CF. Both young humans and ferrets with CF had greatly impaired PP secretion, despite an abundance of islet PP cells and intact PP secretion from perifused CF ferret islets. Pan-neuronal staining in the pancreas was nearly absent from CF humans and of aberrant morphology and distribution in CF ferrets. Parasympathetic neuronal staining was nearly absent in both ferret and human CF pancreata. When exocrine pancreatic disease was delayed until after the first month of life by administering a CFTR modulator in *CFTR^G551D^* homozygous ferrets, there was preservation of pancreatic neuronal markers and restoration of PP secretory responsiveness to oral glucose. We conclude that the CF pancreas is impacted by a loss and remodeling of its innervation, secondary to early-life pancreatic disease, likely contributing to impaired islet hormone secretion.

## Introduction

Despite remarkable advances in clinical care, people with cystic fibrosis (PwCF) continue to experience disproportionately high rates of a distinct but incompletely understood form of diabetes mellitus termed CF-related diabetes (CFRD). Hyperglycemic abnormalities are present by early childhood in many PwCF [1]. By middle age roughly 50% of PwCF have developed CFRD [2]. In PwCF, hyperglycemia and diabetes are primarily driven by inadequate insulin secretion [2]. Especially deficient is early-phase insulin secretion [3]. Even in PwCF who have normal glucose tolerance, early-phase insulin secretion in response to oral glucose is deficient [4]. However, the cellular and molecular underpinnings of this delayed and impaired insulin secretion in cystic fibrosis (CF) have not been fully elucidated.

Other aspects of islet function beyond insulin secretion are also impaired in PwCF. Glucagon secretion in response to hypoglycemia is impaired in PwCF [5], contributing to a propensity for post-glucose load hypoglycemia in PwCF [6,7]. Glucagon suppression by oral glucose may be increasingly impaired in PwCF as glucose tolerance worsens [8,9]. However, the islet hormone with the most substantially impaired secretion is pancreatic polypeptide (PP). In health, the physiological stimuli for PP secretion are feeding and hypoglycemia. In PwCF, the secretion of PP is nearly completely absent both in response to oral glucose and in response to hypoglycemia [5,8].

CF is caused by bi-allelic mutations of the CF transmembrane conductance regulator (CFTR), which encodes an anion channel. CFTR is expressed in multiple organs, including the exocrine pancreas, where it is essential for maintaining proper luminal fluid viscosity and electrolyte composition [10]. Pancreatic exocrine disease begins very early in life in PwCF, with histological loss of exocrine parenchyma often evident in utero by 32–38 weeks post conception [11]. Most PwCF, excepting those with mild mutations, develop clinical exocrine pancreatic insufficiency within the first year of life [12,13]. Exocrine pancreatic insufficiency in PwCF is the main risk factor for diabetes in PwCF [14]. Furthermore, the severity of exocrine pancreatic disease at birth in PwCF predicts the risk of developing CFRD years later [15]. These associations implicate exocrine pancreatic disease in the etiology of impaired insulin secretion in CF, potentially with a special role for early life exocrine pancreatic disease. However, the potential mechanisms for these associations are not entirely clear, especially given that pancreatic islets are relatively spared compared with the marked disruption and loss of exocrine tissue in PwCF [16]. For this reason, mechanisms beyond simple loss of islet mass have been implicated in reducing islet function in PwCF [14].

The highly impaired secretion of PP in PwCF has potential mechanistic implications. In health, the physiological secretion of PP by pancreatic islets is largely driven by vagal parasympathetic efferent innervation to islets [17]. Unlike for PP, the secretion of insulin and glucagon from islets is not primarily dependent upon innervation but rather is primarily regulated by cell-autonomous nutrient sensing [18,19]. Nonetheless, secretion of insulin and glucagon is modulated by islet innervation. Early-phase insulin secretion in response to oral glucose is augmented by vagal parasympathetic efferent innervation to islets [20]. Both the parasympathetic and sympathetic autonomic systems contribute to hypoglycemia-induced glucagon secretion [20]. Taken together, the constellation of islet hormone secretion defects present in PwCF could be consistent with broad loss of autonomic innervation to islets in the CF pancreas.

Rodent-based preclinical models for CF lack exocrine pancreatic disease and CFRD. By contrast, ferrets with CF do develop exocrine pancreatic disease and spontaneous diabetes, which resembles that in PwCF. The pancreas in CF ferrets undergoes predictable decline, passing through age-dependent phases [21,22]. CF ferrets are born with only subtle exocrine pancreatic disease. Then at about 1 month of age, CF ferrets experience a pancreatic crisis consisting of pronounced inflammation and near total loss of acinar tissue. Thereafter, the CF ferret pancreas is characterized by ductal cysts, extensive fibrosis, adipose tissue, and relative sparing of islet mass, phenocopying that which occurs in PwCF.

To better understand whether autonomic islet innervation might be disrupted in CF, we examined neuronal immunostaining in pancreatic samples from both humans and ferrets with CF. Because islets are interspersed within the pancreas, intact autonomic innervation from the central nervous system (CNS) must involve a continuous anatomic path that traverses non-endocrine aspects of the pancreas [20,23]. Thus, our immunostaining approach examined both the endocrine and non-endocrine pancreas. We also examined PP responses to physiologic stimuli in young children with CF and in CF ferrets as a reporter of parasympathetic innervation.

## Methods

### Study approval

The clinical study protocol was approved by the institutional review boards of both the University of Iowa and the University of Minnesota. All participants provided informed consent and assent, as appropriate. All animal experimentation was conducted at the University of Iowa and was approved by the Institutional Animal Care and Use Committee at the University of Iowa (#1071945-035). Blood was collected during brief anesthesia with inhaled isoflurane. Euthanasia was conducted using Euthasol, 150 mg/kg, by intraperitoneal injection.

### Ferret husbandry

Ferrets were housed in separate cages under controlled temperature (20°C–22°C) and long diurnal light cycle (16 h light/8 h dark) with unrestricted access to water and food [24].

CF ferrets were homozygous for disruption of *CFTR* at exon 10 [25]. CF kits were co-reared with a wild-type (WT) littermate from birth, with both receiving identical care including lifelong antibiotic and nutrition support required to maintain the health of CF ferrets [25]. CF ferret pancreas phases and approximate ages were defined as follows: phase I, pre-crisis, age 5–15 days; and phase II, crisis, 26–60 days; phase III, post-crisis, 75–110 days; phase IV, hyperglycemic risk, 130 days and beyond [25]. The co-reared WT ferrets are referred to as control (CON) in the present article. In some experiments, non-co-reared WT ferrets are used and referred to as WT.

Ferrets homozygous for the *CFTR^G551D^* knock-in allele [26,27] were reared on CFTR modulator (VX-770) to delay the onset of overt CF disease until after 30 days of age. *CFTR^G551D^* ferrets were reared as described [27], except as follows. Pregnant jills carrying *CFTR^G551D^* offspring were administered 20 mg/kg VX-770 twice daily on gestational days 28 until 23 days past parturition. Thereafter, VX-770 administration was switched directly to the kits at 10 mg/kg VX-770 twice daily until 30 days of age. In the present article, *CFTR^G551D^* ferrets reared in this manner are abbreviated as CF_delay_ to indicate the delay of overt CF disease.

### Human studies

Human study participants were from a described study, though recruitment continued after the initial publication [28]. Briefly, children aged 3 months through 6 years were prospectively recruited for frequently sampled oral glucose tolerance testing (OGTT) at the University of Iowa and University of Minnesota. Both children with CF and CON without CF were included. The glucose dose for the frequently sampled OGTT was 1.75 g/kg, with a maximum dose of 75 g [28]. The study protocol, including weight-based fasting times and inclusion/exclusion criteria, has been published [28]. A total of 62 OGTTs were performed in 38 participants. Of these, 27 underwent a single OGTT, 7 two OGTTs, 2 three OGTTs, 1 four OGTTs, and 2 six OGTTs. Six OGTTs did not include samples for all time points and were excluded from the area under the curve analysis. All OGTT data were included in linear mixed effect modeling analysis, treating subject ID as a random effect accounting for the repeated nature of the OGTTs in some individuals.

### Glycemic tests in ferrets

The oral glucose dose for ferret OGTT was 1.75 g/kg, administered after a 4-h fast [25]. Mixed meal tolerance tests (MMTTs) were also performed after a 4-h fast, administering a slurry of canned food and an elemental liquid diet (Elecare, Abbott Laboratories) with the amount normalized to body surface area [22]. Blood glucose was measured by a portable meter (One Touch Lifescan). Hyperglycemic-to-hypoglycemic clamps were performed in 4-h-fasted conscious ferrets using percutaneous venous catheters [25,29]. At time 0, dextrose was infused at 50 mg/kg·min, with the rate adjusted thereafter with a glycemic goal of 240 mg/dl. At 60 min, the glucose infusion was discontinued. A loading dose of insulin lispro (Eli Lilly) of 50 mU/kg was infused over 5 min, and a continuous infusion of 20 mU/kg/min was initiated [29]. A variable dextrose infusion was resumed as hypoglycemia ensued with a glycemic goal of 45 mg/dl.

### Neonatal islet isolation and perifusion

Islets were isolated from neonatal ferrets and matured in culture over 9 days as described [22]. Islet perifusion assays [21] were performed in a closed small-volume chamber after equilibration with perifusion buffer of 1.67 mM glucose/KRB at 38.5°C for 30 min. Islets were then stimulated with perifusion buffer containing 10 mM arginine. Islet PP content was quantified at the end of each experiment.

### Measurement of hormone analytes

Plasma PP and glucagon were measured using a Human Metabolic Hormone Magnetic Bead Panel (EMD Millipore Cooperation, Billerica, MA).

### Immunomicroscopy of human and ferret pancreas

Tissue samples embedded in paraffin were sectioned 10 μm thick unless otherwise noted. Fixed human pancreatic tissue was obtained from the National Disease Research Interchange and paraffin blocks embedded at the University of Iowa Comparative Pathology Laboratory. Slides were deparaffinized sequentially with xylene thrice, 100% ethanol twice, 95% ethanol once, and 70% ethanol once, and then rinsed in distilled water. Antigen retrieval was in citrate buffer, pH 6.0, 110°C for 15 min. After washing with distilled water once and phosphate-buffered saline (PBS) thrice, sections were permeabilized in 0.2% Triton X-100 for 15 min. After two washes in PBS, the tissue was blocked with 5% normal goat serum (NGS) for 1 h. After blocking, primary antibodies were added in 5% NGS/PBS and incubated overnight at 4°C. The next day, the slides were washed thrice in PBS before incubation with secondary antibodies diluted 1:300 in PBS for 2 h. The tissue slide was then washed three more times for 10 min each in PBS before confocal microscopy. *In situ* hybridization was performed on paraffin sections as described [25].

Whole-mount ferret pancreas tissue was excised fresh in approximate 5 × 5 × 1 mm samples. Tissues were immediately fixed in 4% paraformaldehyde for 24 h at 4°C, washed twice in PBS, and then 3 days at 4°C in blocking buffer (19% donkey serum, 0.05% Triton X-100, 1 mM CaCl_2_ in PBS). Antigen retrieval occurred in citrate buffer, pH 6.0, at 55°C for 24 h. After three washes in PBS, primary antibodies were added in diluent buffer (same as blocking buffer except 1% donkey serum) for 3 days at 37°C. After three washes in PBS, the tissue was incubated with secondary antibodies (1:300) and DAPI (1:10000; ThermoFisher) in PBS for 2 days at 37°C. After three PBS washes, the sample was optically clarified with a tissue-clearing solution (Ce3D, Biolegend, catalog #427704) for 2 h.

Primary and secondary antibodies employed are listed in Supplementary Table S1. Images were acquired on a Zeiss LSM 710 confocal microscope with Zen 2010 software by personnel blinded as to the hypotheses under study. Specificity of immunofluorescence staining was ascertained by comparing images from CON samples to known expected staining patterns [30,31]. Negative controls included secondary only and cross-primary/secondary pairings for TUJ1 and VAChT staining. Image files were processed using Fiji:ImageJ [32]. No non-linear processing was used. Confocal image stacks are shown as maximum intensity projections unless otherwise noted. Quantification of neuronal density was measured on blinded images and expressed as neuronal area normalized to pancreas area.

### Sex as a biological variable

For both humans and ferrets in our study, both male and female participants/animals/samples were used. For analysis, sex was considered as a biological variable where noted.

### Statistics

Graphical data are presented as dot plots or as mean ± standard error of measurement. All statistical analysis was performed in R (version 4.5.0, R Foundation for Statistical Computing, Vienna, Austria). The statistical significance of pair-wise differences was assessed by the Wilcoxon rank-sum test after averaging repeated measures. For data containing repeated measures, statistical significance was assessed by mixed effects modeling using the *lme4* R package [33]. Log transformation was applied when this increased normality of the residuals. For cases demonstrating a significant omnibus group interaction with time, post-hoc testing was performed employing the *emmeans* R package, using the Kenward–Roger method to assess degrees of freedom and Tukey's method to adjust for multiple testing. *P*-values less than 0.05 were considered significant.

## Results

### Diminished general innervation in human and ferret CF pancreas

To assess the impact of CF on pancreatic neuronal innervation, human and ferret pancreata were immunostained using the pan-neuronal neuron-specific class III beta-tubulin “TUJ1” antibody. Images from human pancreata are shown in Figure 1A and Supplementary Figure S1A, which shows only the grayscale of TUJ1 staining for easier identification of neuronal structures. In the human CON pancreas, there were abundant TUJ1+ neuronal axons penetrating the non-endocrine pancreas and interfacing with islets. As previously described [34,35], beta-cells in the human CON pancreas exhibited a degree of TUJ1 staining. In human CF pancreas, few TUJ1+ structures were observed, with nearly none exhibiting morphology clearly consistent with neuronal or axonal identity. Ferrets were examined in age phase III, after exocrine damage had occurred and by which time fibrosis had become extensive in the CF pancreas. In ferret pancreas (Figure 1B and Supplementary Figure S1B for grayscale of TUJ1 staining only), there were scattered TUJ1+ neuronal axons in the WT non-endocrine pancreas including some that interfaced with islets. Additionally, within islets of WT ferrets there were networks of TUJ1+ axonal processes. In CF ferret pancreas, the islets also contained a network of TUJ1+ axonal-appearing structures. However, these exhibited a different morphology than in WT islets, including many processes in and near the CF islets that appeared thicker than the axons in WT islets (higher magnification shown in Supplementary Figure S2). To explore the potential developmental origins of the neuronal disruption in the CF pancreas, we examined newborn ferret pancreata (Supplementary Figure S3). Both WT and CF newborn pancreata contained plentiful TUJ1+ cell bodies and axons, in contrast with the absence of TUJ1 positivity observed in CF ferrets by phase III. The intra-islet TUJ1 staining plexus apparent in older ferret islets was not clearly present in the newborn pancreas islets, indicating that additional neuronal development occurs after the newborn period in ferrets as relates to islets. The previously described [22] smaller islets in CF newborn pancreas were apparent in the images.

**Figure 1:**
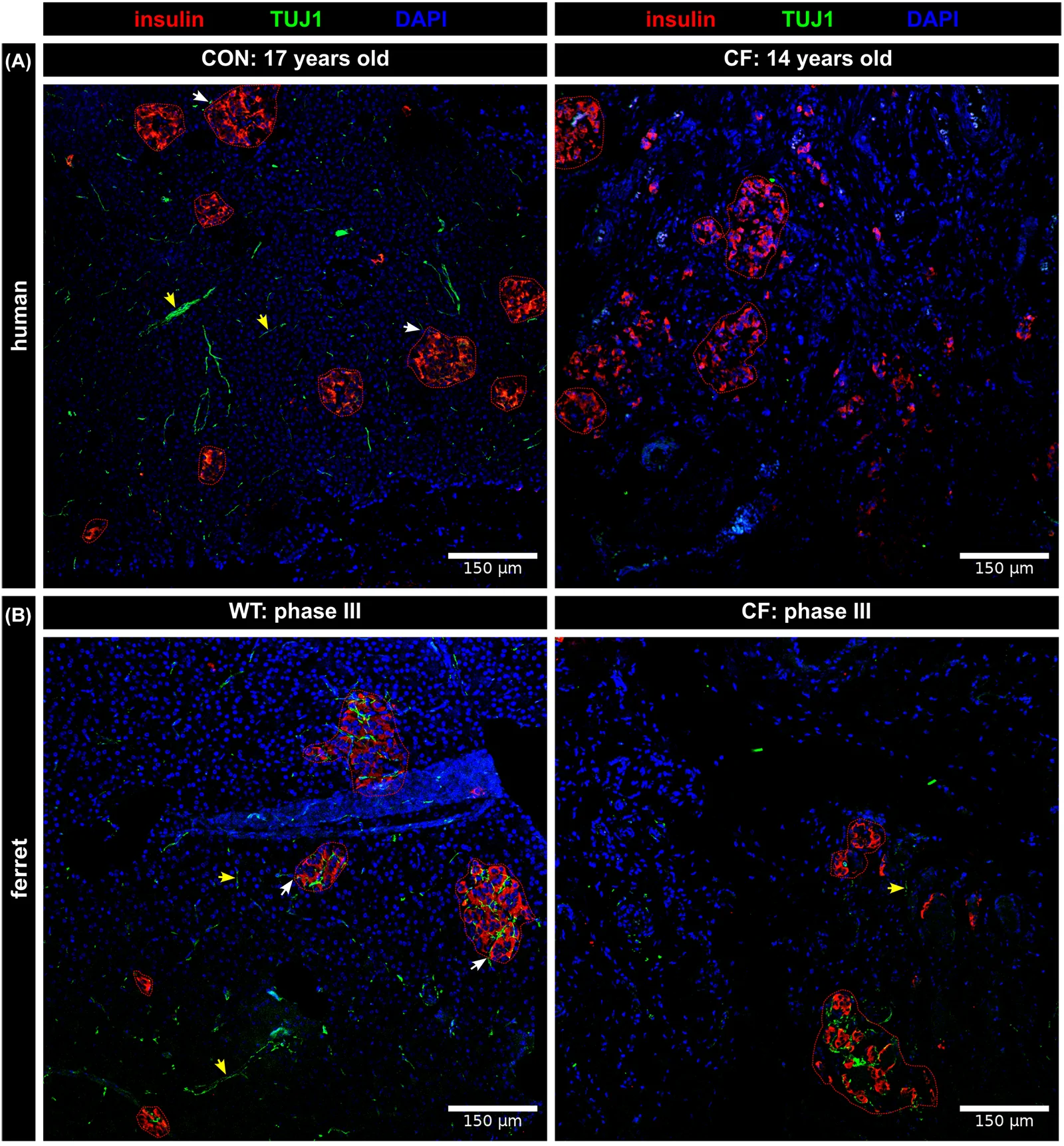
Pan-neuronal pancreatic innervation Class III beta-tubulin (TUJ1, green) and insulin (red) co-staining of human (**A**, top row) and ferret (**B**, bottom row) pancreas on 10 μm paraffin sections. Blue is DAPI. Left column: CON human, WT ferret; right column: CF. Ages are indicated in the figure. Human micrographs representative of three independent images. Ferret micrographs are representative of over 10 images collected from two animals. Supplementary Figure S1 shows grayscale images of the TUJ1 channel from these micrographs. Beta-islets are circled with red dotted lines, though small and irregular islets are not marked. Examples of TUJ1+ axonal processes are shown with yellow arrows. Examples of TUJ+ axons adjacent or interfacing with islets are highlighted with white arrows.

### Diminished parasympathetic innervation of the CF pancreas

PP secretion is mediated specifically by parasympathetic innervation. We thus examined the parasympathetic neuronal marker, vesicular acetylcholine transporter (VAChT), by immunostaining in human and ferret pancreata. To establish the validity of VAChT immunostaining in ferret pancreas, we examined VAChT:TUJ1 localization in WT sections. Like in other species [30], VAChT immunostaining of WT ferret pancreatic neurons coincided with TUJ1-positive neurons (Supplementary Figure S4). VAChT immunostaining exhibited punctate patterning along axons, consistent with its localization to axonal varicosities, whereas TUJ1 immunostaining was continuous along axons, consistent with these patterns in other species [30] (Supplementary Figure S4). Images from human pancreata are shown in Figure 2A and Supplementary Figure S5A, which shows VAChT staining in grayscale. In the human CON pancreas, VAChT+ axons were found scattered throughout much of the non-endocrine parenchyma, including near islets. By contrast, in human CF pancreas samples, few if any VAChT+ cellular structures could be identified. Images from four separate CF and four separate CON human pancreata are shown in Supplementary Figure S6. In one of the CF pancreata, occasional VAChT+ neurons/axons were identified. Images from ferret pancreata are shown in Figure 2B and Supplementary Figure S5B, which shows VAChT staining in grayscale. In phase III ferrets, the WT pancreas contained scattered VAChT+ axons in the non-endocrine parenchyma, occasionally in close proximity to islets. By contrast, in CF pancreas, VAChT-positive cellular structures were difficult to locate and only rarely observed. To investigate the early-life origins of the neuronal disruption in the CF pancreas, we examined VAChT content in newborn ferret pancreas (Supplementary Figure S7). The newborn WT pancreas exhibited abundant scattered VAChT axonal processes and possibly cell bodies. Unexpectedly, newborn CF pancreata exhibited even greater VAChT expression compared with WT, including numerous cell-sized structures.

**Figure 2:**
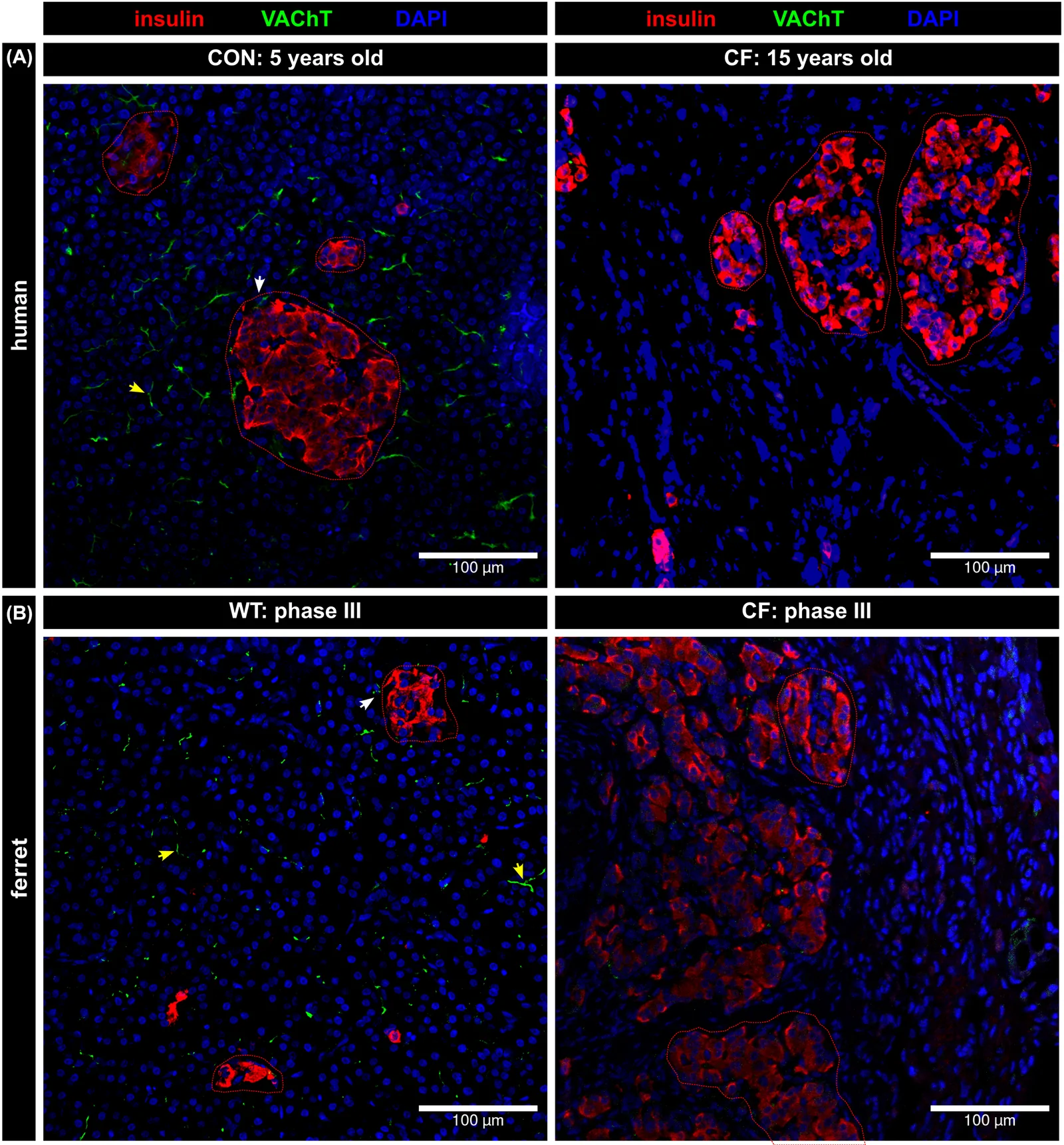
Pancreatic parasympathetic innervation VAChT (green) and insulin (red) co-staining of human (**A**, top row) and ferret (**B**, bottom row) pancreas on 10 μm paraffin sections. Blue is DAPI. Left column: CON human, WT ferret; right column: CF. Ages are indicated in the figure. Supplementary Figure S5 shows grayscale images of the TUJ1 channel from these micrographs. Human micrographs from four separate pancreata per group are shown in Supplementary Figure S6. Ferret micrographs are representative of images collected from 6+ separate pancreata per genotype. Beta-islets are circled with red dotted lines, though small and irregular islets are not marked. Examples of VAChT+ axonal processes are shown with yellow arrows. Examples of VAChT+ axons adjacent or interfacing with islets are highlighted with white arrows.

### Diminished PP secretion caused by cystic fibrosis

Parasympathetic innervation mediates the stimulation of PP secretion. To determine whether CF impacts parasympathetic ability to stimulate PP secretion, we examined samples from young children and young ferrets who underwent OGTT. The study design and patient characteristics have been previously published for the human [28] and ferret [25] studies, though these new data include additional subsequently collected samples. Children with CF exhibited significantly lower PP levels than CON (Figure 3A), with significantly lower incremental area under the curve for the 0–30 min peak (iAUC_30_) (Figure 3B). Only CON children, but not children with CF, exhibited a significant increase in PP levels over baseline during oral glucose (Figure 3A). The CF participants consisted of 11 females and 11 males, whereas CON were 8 males and 2 females. When sex was included in the linear model, it did not significantly influence PP levels directly or via interaction with group. The children with CF whose CFTR mutations are known to retain residual function are not included in Figure 3, but rather are shown separately in Supplementary Figure S8. Interestingly, the PP levels in those children with residual CFTR function were more similar to CON than to those without residual CFTR function. Like humans, young CF ferrets exhibited lower stimulated PP levels than CON in response to oral glucose (Figure 3C) and had significantly lower iAUC_30_ than CON (Figure 3D). Both groups consisted of seven females and two males. When sex was included in the linear model, it did not significantly influence PP levels directly or via interaction with group. Unlike for the glucose challenge, PP levels did increase in response to mixed meal testing in CF ferrets indistinguishable from CON (Supplementary Figure S9).

**Figure 3:**
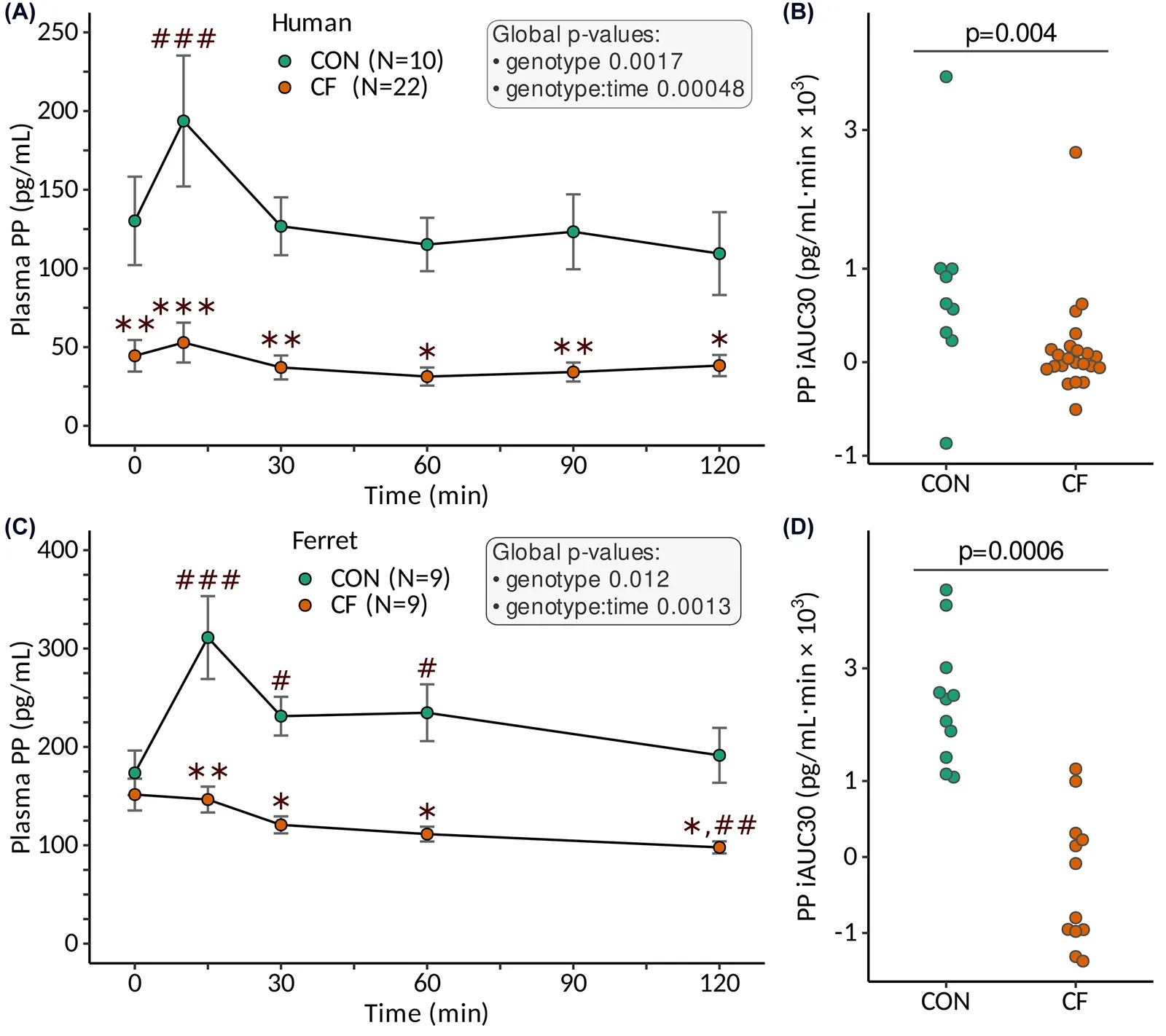
PP levels in response to OGTT Serum PP levels in young humans (**A, B**) and ferrets (**C, D**). Time course (A, C) and incremental AUC for the first 30 min (B, D, iAUC_30_) are shown. The studies included repeated measures; for this reason, *n* represents the number of independent participants rather than the number of tests examined. Human participants with CF that harbored CFTR mutations with residual function are not included. (A, C) Significance was determined by mixed effects modeling with the arising global *P*-values related to genotype details in insets. Post hoc *P*-values are indicated by *P<0.05, **P<0.005, and ***P<0.0005 for the difference between CF and CON, and #*P<*0.05, ##*P<*0.005, and ###*P<*0.0005 for the difference from baseline for that specific genotype. (B, D) Significance was determined by the Wilcoxon rank sum test, averaging iAUC30 values for participants with repeated tests but displaying all test values.

Another physiologic stimulus for the secretion of PP is hypoglycemia. Adult PwCF exhibit impaired PP secretion in response to hypoglycemia [5]. In our study, induction of hypoglycemia was not performed in children but was performed in ferrets as part of a hyperglycemic-to-hypoglycemic clamp (Figure 4A) to interrogate islet function [25]. Throughout the clamp, CF ferrets exhibited lower PP levels than CON ferrets (*P* = 1.1e−10; Figure 4B). Similarly, glucagon secretion in CF ferrets was significantly less compared with CON (*P* = 3.4e–8; Figure 4C).

**Figure 4:**
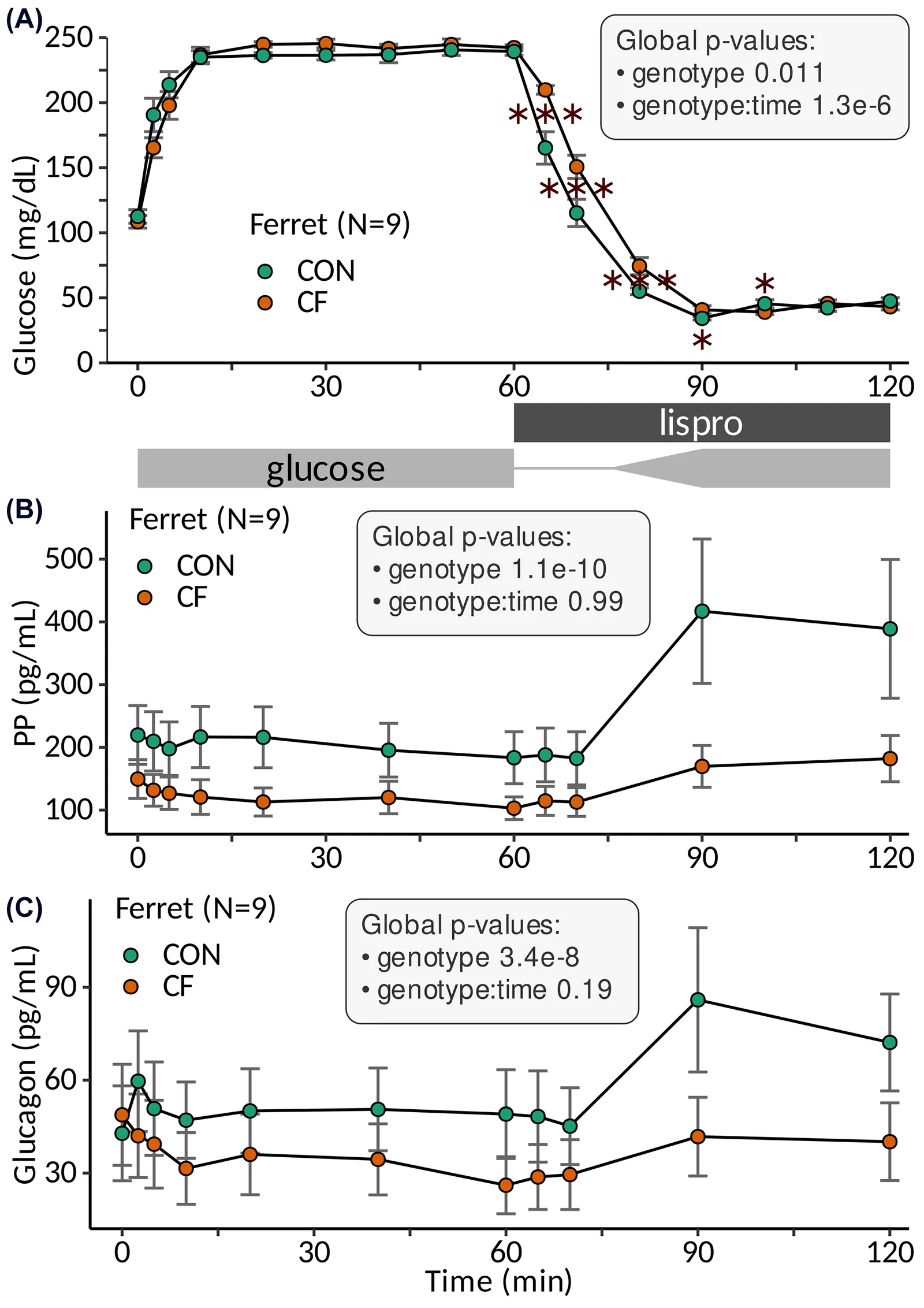
Hyperglycemic-to-hypoglycemic clamp of CF versus CON ferrets Timeline indicates variable glucose infusion to achieve hyperglycemia 0–60 min and then re-initiates as needed after 60 min to maintain a glucose goal of 45 mg/dl. The timeline also indicates initiation of insulin lispro infusion at 60 min to induce hypoglycemia. The resultant glucose, PP, and glucagon levels are shown (**A**–**C**), respectively. The glucose, glucose infusion rates, and insulin data for 0–60 min for six of the ferrets shown per genotype have been published [25]. Ferrets ranged from 63 to 446 days in age and were paired by age. Significance was determined by mixed effects modeling with the arising global *P*-values related to genotype details in insets. Post hoc *P*-values are indicated by *P<0.05, **P<0.005, and ***P<0.0005 for the difference between CF and CON. There were numerous post hoc significant changes from baseline for both genotypes in panel (A), but these are plainly evident, and statistical symbols are omitted for visual clarity.

### Pancreatic polypeptide cells are present in the CF pancreas

To better understand the cause of low PP levels in CF, we immunostained the pancreas of humans and ferrets for PP cells. In the human CON pancreas, pancreatic PP cells were scattered among some islets in the pancreas, as expected (Figure 5A and Supplementary Figure S10A). By contrast, PP cells appeared more abundant in human pancreas with CF, including those with CFRD (Figure 5B,C and Supplementary Figure S10B,C). In CON ferret pancreata, as in CON humans, only scattered cells expressed PP (Figure 6, left panels). By contrast, PP-expressing cells appeared more frequently in the pancreas of CF ferrets (Figure 6, right panels). These results are consistent with published findings in ferrets of increased PP mRNA starting at phase II [23] and increased abundance of PP cells in human CF pancreas [16].

**Figure 5:**
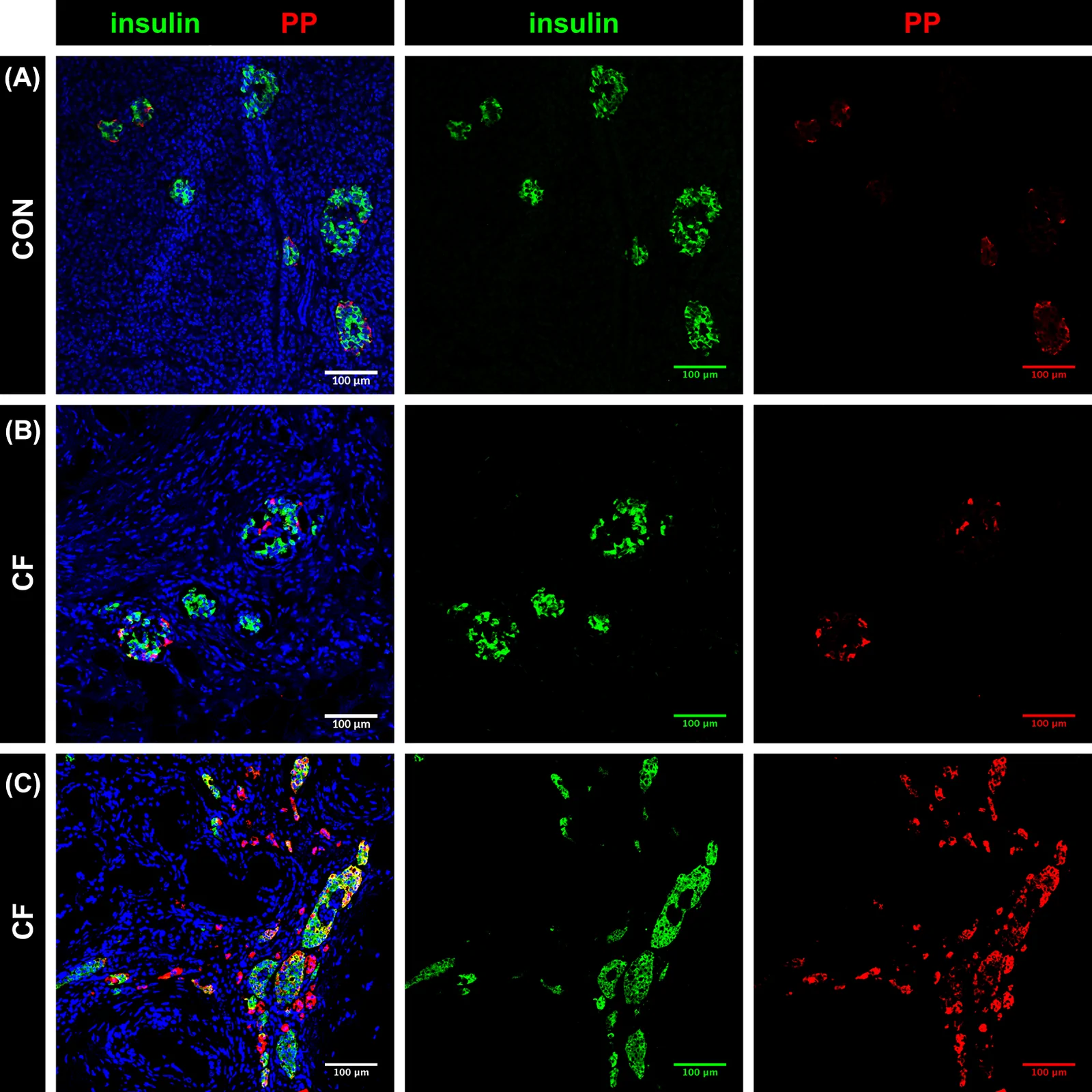
Immunostaining of PP- and beta-cells in human pancreata Each row represents a different individual without (**A**, CON) or with CF (**B**,** C**). Green: insulin. Red: PP. Blue: DAPI. Middle and right columns show the isolated insulin and PP channels. Scale bars are 100 μm. Human CF micrographs are representative of images collected from five separate CF pancreata. Images with greater magnification can be found in Supplementary Figure S10.

**Figure 6:**
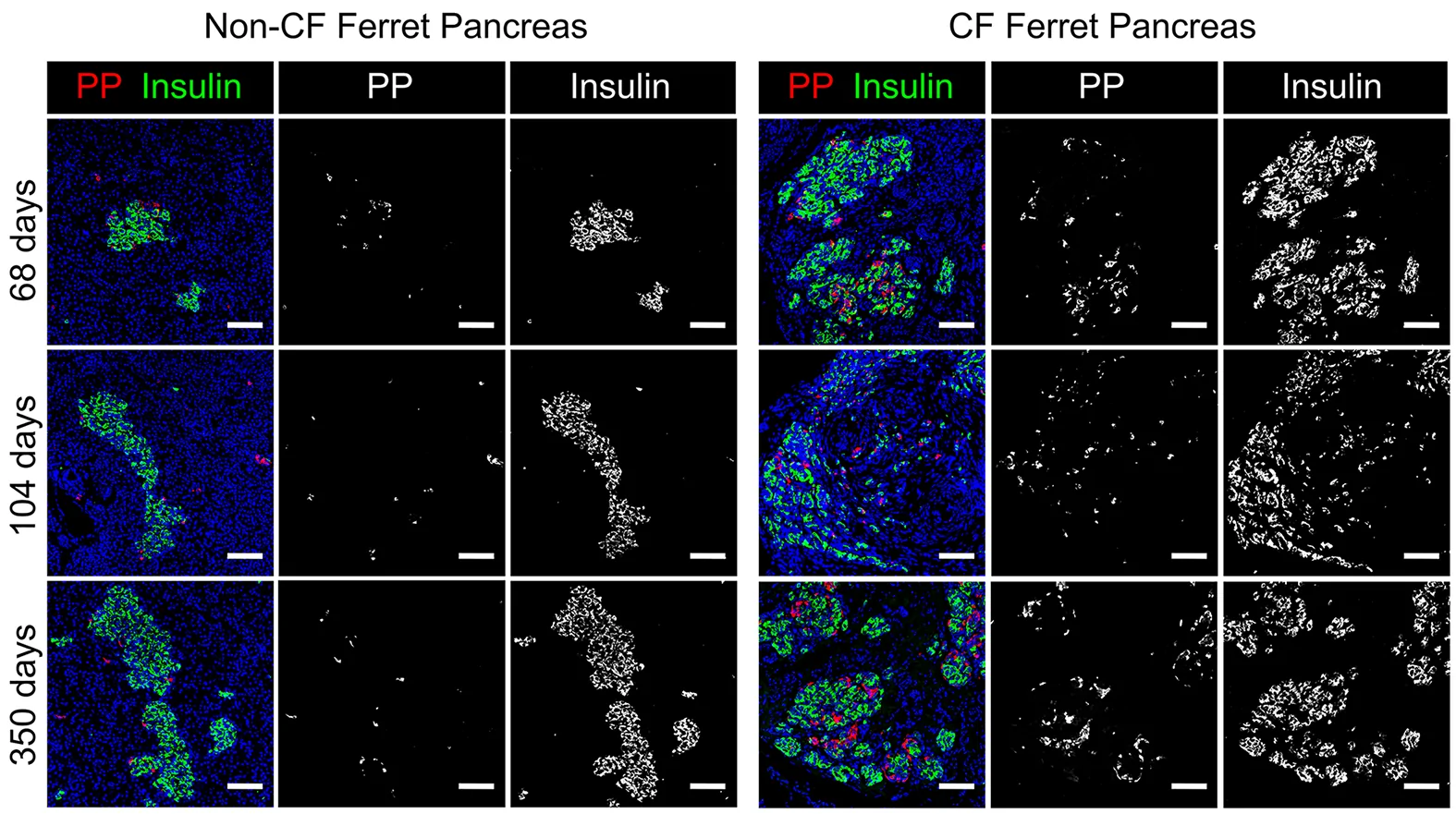
Immunostaining of PP- and beta-cells in ferret pancreata Left panels, CON (non-CF) pancreata; right panels, CF pancreata. Green: insulin. Red: PP. Blue: DAPI. Scale bars represent 100 μm.

### Cystic fibrosis ferret islets are capable of pancreatic polypeptide secretion

As a next step in understanding the failure of PP to be secreted in CF, we examined isolated cultured ferret islets. To determine whether CF islets were capable of secreting PP, we used arginine as a secretion stimulus [36]. In a perifusion system, arginine rapidly stimulated PP secretion from islets into the media (Figure 7). PP secretion did not differ between CF and CON islets when expressed either as absolute concentration or as % secretion relative to islet PP content (Figure 7A,B). PP secretion was lower in CF versus CON islets when expressed as a fold change from baseline (Figure 7C). One CON islet preparation was an outlier in that it secreted roughly a magnitude of order more PP than the other CON islets, including at baseline. Data from this outlier was not included in Figure 7. In the interest of transparency, the outlier is included in Supplementary Figure S11, as evidenced by the large CON error bars. When this outlier was included, CF and CON PP secretion when expressed as absolute and % secretion did not differ globally or at any specific time point (Supplementary Figure S11A,B). When expressed as fold change from baseline, multiple time points for CF exhibited less relative PP secretion than CON (Supplementary Figure S11C). Islet PP content did not differ between the genotypes (details in Figures 7 and Supplementary Figure S11 legends).

**Figure 7:**
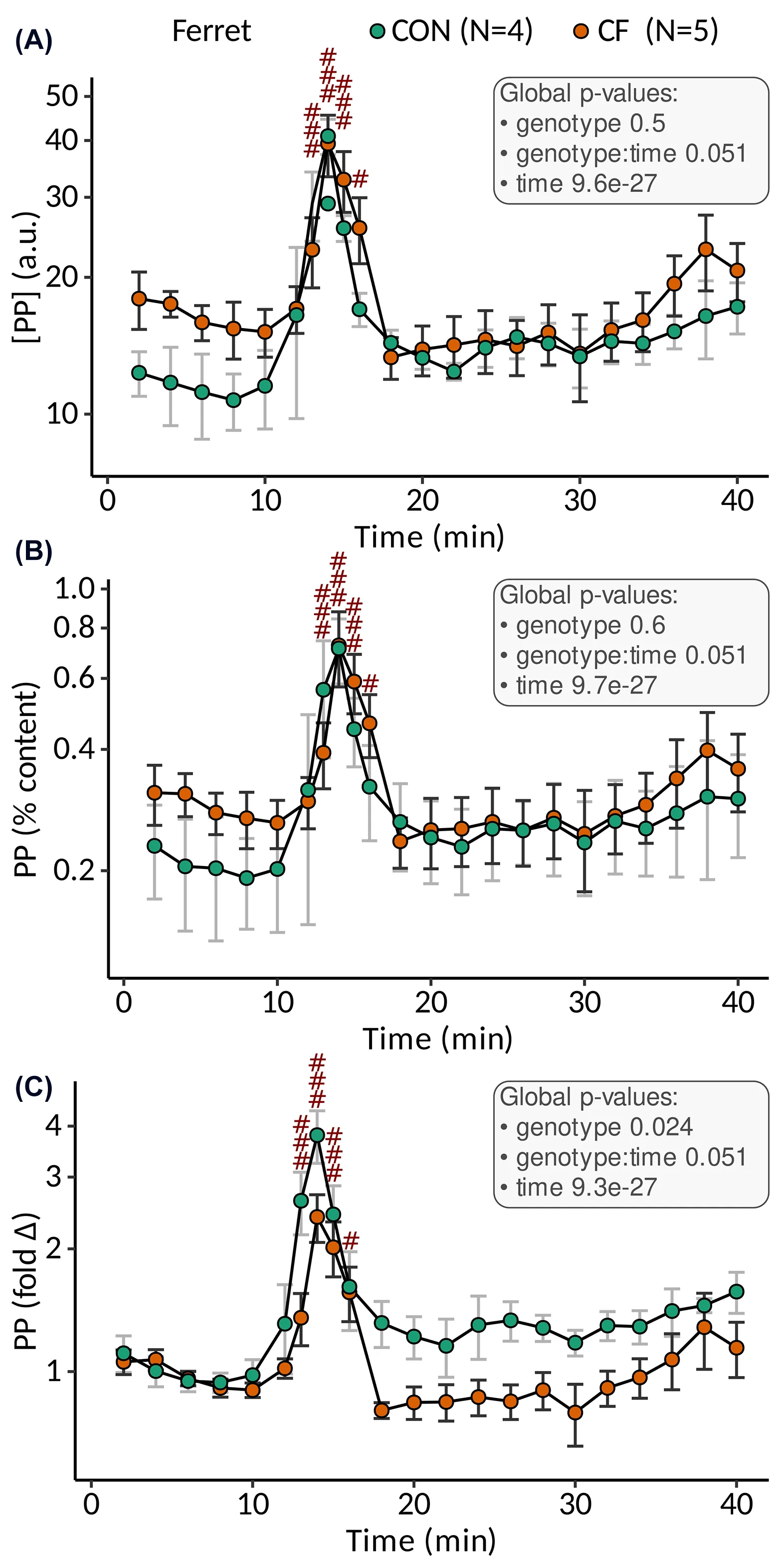
Perifusion of islets Arginine was introduced into the perifusate at 10 min. The single outlier CON prep that secreted one magnitude-of-order greater PP than other preparations is omitted. Analysis without omitting this outlier prep can be found in Supplementary Figure S11. (**A**) Perifusate PP concentration. (**B**) Fractional secretion compared with post-perifusion islet PP content. (**C**) Fold change compared with the mean 0–8 min baseline. Significance was determined by mixed effects modeling on log-transformed data with the arising global *P*-values shown in the insets. Post hoc *P*-values for joint change from the initial four baseline values are indicated as # *P<*0.05 and vertically stacked ### *P<*0.0005. Islet PP content did differ between groups (CON: 6924 ± 2202 pg/islet equivalent; CF: 5943 ± 734; *P* = 0.73).

### Sympathetic innervation of the CF pancreas

Sympathetic innervation modulates glucagon and insulin secretion [20]. Sympathetic nerves can be localized by immunostaining for tyrosine hydroxylase. Images from human pancreata are shown in Figure 8A and Supplementary Figure S12A, which shows the grayscale of tyrosine hydroxylase staining only. In human CON pancreas, there were occasional tyrosine hydroxylase-positive axonal processes traversing within the non-endocrine parenchyma near islets. In the CF human pancreas, very few tyrosine hydroxylase-positive neuronal structures were identified. Images from ferret pancreata are shown in Figure 8B and Supplementary Figure S12B, which shows the grayscale of tyrosine hydroxylase staining only. The ferret pancreas exhibited a different staining pattern for tyrosine hydroxylase than in humans. In WT ferrets, islets exhibited a network of axonal, tyrosine hydroxylase-positive processes. Occasional insulin-expressing cell bodies were also tyrosine hydroxylase positive (Figure 8B). There were occasional tyrosine hydroxylase-positive axonal processes in the non-endocrine parenchyma. By contrast, in the CF pancreas, there were few if any tyrosine hydroxylase-positive axons. However, many islet cell bodies were positive for tyrosine hydroxylase and insulin (Figure 8B and Supplementary Figure S12B). A higher fraction of insulin-positive cells also expressed tyrosine hydroxylase in CF than in CON pancreas (CF: 37% ± 3%; CON: 5% ± 2%; *P* = 0.0008; at least three sections from three pancreata per group). There was no overlap of tyrosine-hydroxylase-positive cells with cells expressing glucagon, PP, or somatostatin in WT or CF pancreata (Supplementary Figure S13). That beta-cells can express tyrosine hydroxylase has been previously observed [37], as will be expounded upon in discussion.

**Figure 8:**
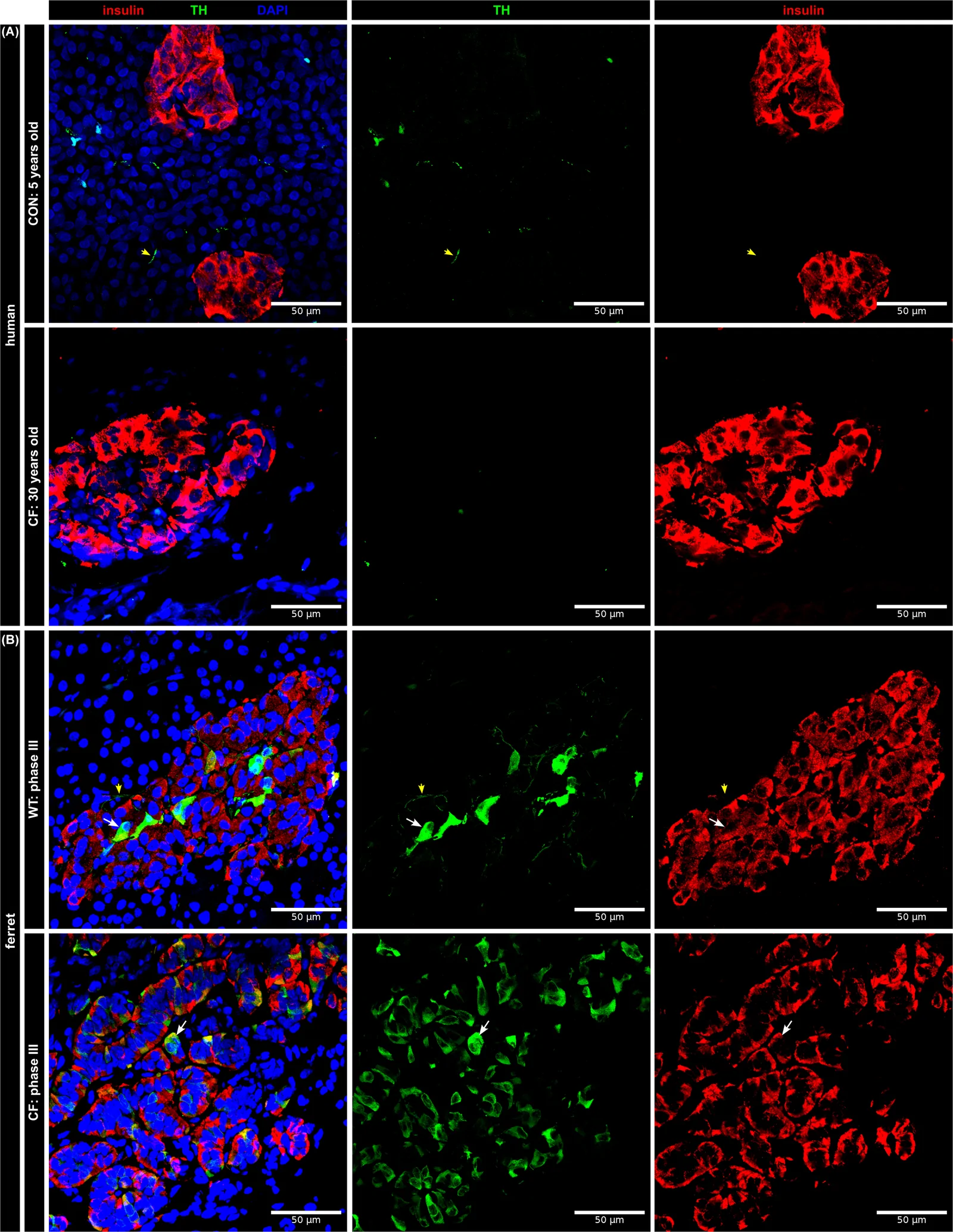
Tyrosine hydroxylase immunostaining Samples were from human (**A**) and ferret (**B**) pancreas. Green = TH; Red = insulin; Blue = DAPI. Ages and group are indicated in the figure. Human micrographs representative of three independent images from one pancreas. Ferret micrographs are representative of images from three WT and two CF pancreata, each undergoing at least six independent images. Yellow arrows indicate example TH+ axons. White arrows indicate example TH+ insulin+ cells. The tyrosine hydroxylase channel is shown in Supplementary Figure S12 using grayscale with an elevated background to allow full examination.

### Partial amelioration by delay of CF pancreatic disease

The above data indicate that, although CF ferret pancreata contain abundant neuronal components at birth, there is a loss and/or deformation of these elements by the third month of life. Importantly, pancreatic innervation of islets undergoes substantial organization and maturation in the postnatal period [38]. These points raise the possibility that the perinatal period represents a critical age of susceptibility to long-lasting disruption of pancreatic innervation. To investigate this, we utilized ferrets harboring *CFTR^G551D^* knock-in [26,27]. This mutation inactivates CFTR function in a manner that is allosterically restored by the CFTR modulator VX-770. VX-770 is a small molecule used to treat PwCF (pharmaceutical name ivacaftor) who have *CFTR^G551D^* and related mutations. Continuous exposure of *CFTR^G551D^* ferrets to VX-770 starting prenatally during the last third of pregnancy prevents exocrine pancreatic disease [27]. However, when VX-770 is discontinued, exocrine pancreatic disease immediately initiates. We examined the pancreata of *CFTR^G551D^* ferrets who were maintained on VX-770 until 30 days of age, then taken off VX-770 to allow overt CF pancreatic disease to ensue. The *CFTR^G551D^* ferrets reared in this manner are abbreviated CF_delay_ in the present article. We then examined pancreata from CF_delay_ ferrets at age 75 days. Representative resultant images are shown in Figure 9 and Supplementary Figure S14, which shows the grayscale of neuronal staining only. Whole-mount micrographs identified a plethora of TUJ1+ immunostaining neurons in the pancreata of WT ferrets. In CF_delay_ ferret pancreata, there was even more copious TUJ1+ immunostaining neuronal structures (Figure 9A and Supplementary Figure S14A); total TUJ1+ neuronal density was increased in CF_delay_ compared with WT pancreas (*P<*0.05, WT: 3.3 [standard error range: 1.7–6.5]%; CF_delay_ 17.2 [11.3–26.3]% area; *n* = 26, 25 sections from 2, 5 pancreases examined, respectively). Animated dimensional renderings of example WT and CFdelay thick section micrographs are found in Supplementary material. The tightly clustered islets in the CF_delay_ pancreas demonstrated the loss of exocrine tissue from having been taken off of VX-770. The neuronal abundance in CF_delay_ pancreas was also observed in thin sections (10 μm; Supplementary Figure S15). VAChT immunostaining neuronal structures were found scattered throughout the pancreas of both WT and CF_delay_ ferrets (Figure 9B and S14B). Consistent with expectations, VAChT immunostaining co-localized with TUJ1+ in CF_delay_ ferrets (Supplementary Figure S16). To test the functional consequences of delaying pancreatic disease, we examined oral glucose-stimulated PP secretion in 9-month-old CF_delay_ ferrets that had been taken off VX-770 at 4 months old. There was no difference in PP levels between the CF_delay_ and CON ferrets (Figure 10A), including a nearly identical peak PP level at 30 min. Likewise, the PP iAUC_60_ was not statistically different between the two groups (Figure 10B).

**Figure 9:**
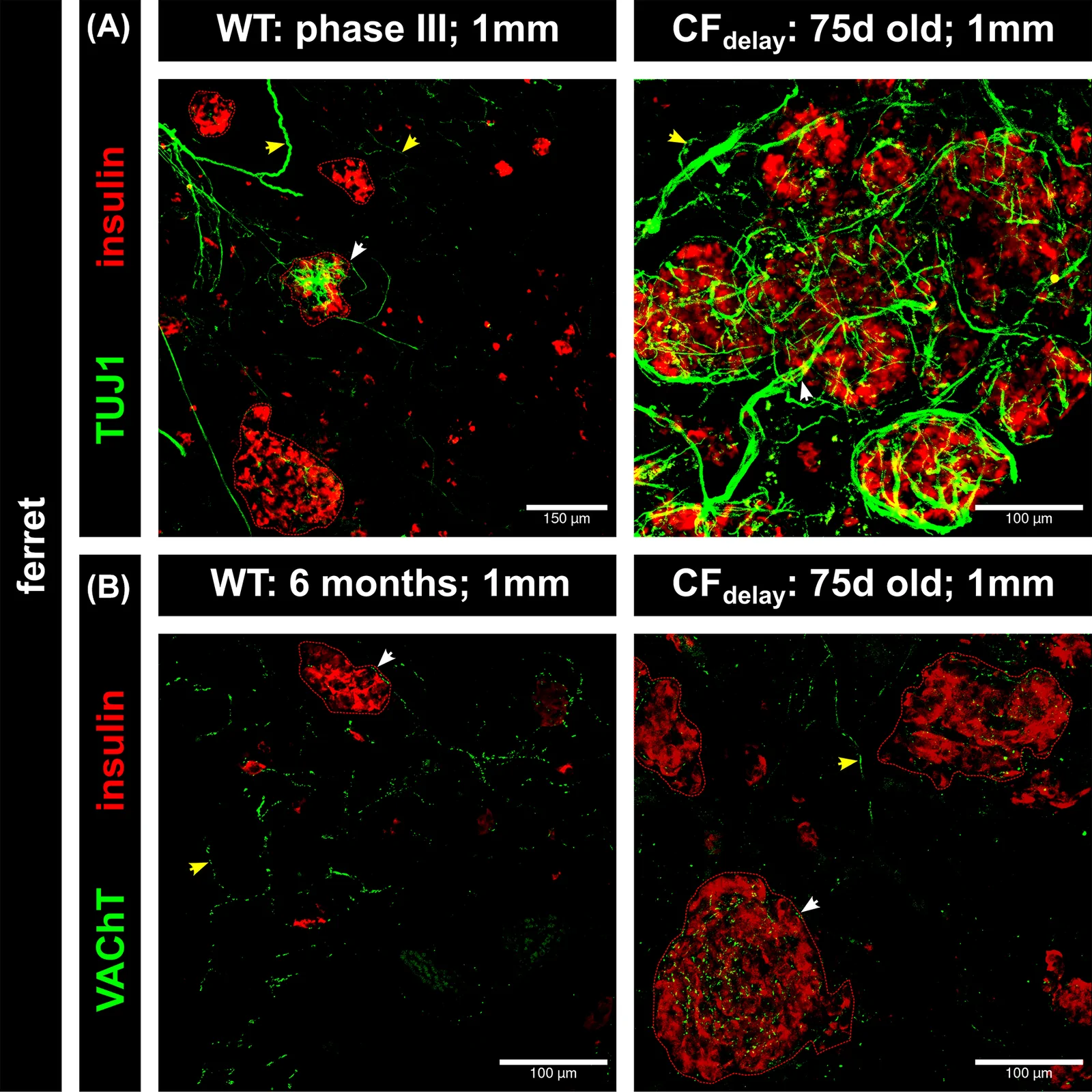
Delay of CF disease spares subsequent pancreatic neuronal innervation CF_delay_ ferrets harboring *CFTR^G551D^* were maintained on VX770 until 30 days old. CF_delay_ pancreata were sampled at 75 days of age. Red = insulin. WT (left column) and CF_delay_ ferrets (right column). (**A**) Top row, pan-neuronal marker TUJ1 in 1 mm whole-mount pancreas. Green = TUJ1. (**B**) Bottom row, VAChT immunostaining on 1 mm whole-mount pancreas. Green = VAChT. WT micrographs are representative of at least five independent images; CF_delay_ micrographs representative of images from five pancreata. Beta-islets are circled with red dotted lines, though small and irregular islets are not marked, nor are islets in the top right, as islets occupy most of the figure. Examples of TUJ1+ axonal processes are shown with yellow arrows. Examples of TUJ+ axons adjacent or interfacing with islets are highlighted with white arrows.

**Figure 10:**
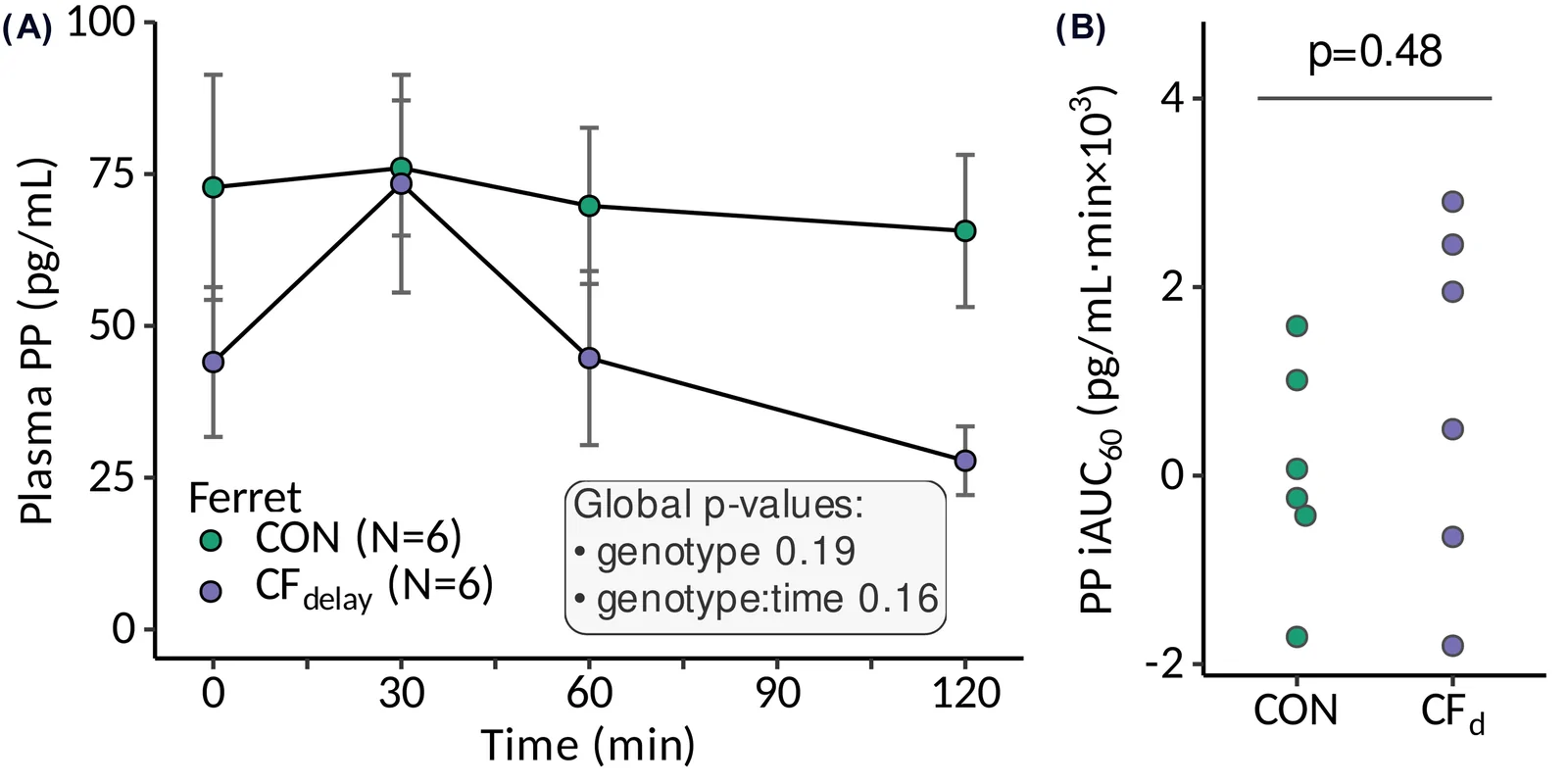
PP levels during OGTT in CON and CF_delay_ ferrets at age 9 months The CF_delay_ ferrets had been taken off of VX-770 at 4 months of age. (**A**) Time course. (**B**) Incremental area under the curve between 0 and 60 min (iAUC_60_) for CON and CF_delay_ (CF_d_). (A) Significance was determined by mixed effects modeling with the arising global *P*-values related to genotype details in insets. (B) Significance was determined by the Wilcoxon rank sum test.

## Discussion

The rich innervation of the endocrine pancreas and the impact of the CNS on glycemia have been recognized since the mid-19th century [30,39]. Islets are innervated by the parasympathetic, sympathetic, sensory, and enteropancreatic neuronal systems [23,30]. Furthermore, neuronal activity modifies hormonal secretion from islets [23]. Here, we provide evidence suggesting endocrine pancreas innervation disruption by CF. Furthermore, we find associated changes in islet hormone secretion that can be caused by loss of innervation.

Our data show extensive morphologic changes to neuronal innervation in the CF pancreas in both humans and ferrets. Early in life, beginning in utero, the human CF pancreas undergoes extensive exocrine damage, such that much of the exocrine pancreatic parenchyma is eventually destroyed. During this process, nearly all acinar tissue is permanently lost. Because acinar cells comprise ∼80% of pancreas mass [40], this leads to a contraction of the spaces between islets. If all the nerves and axons survived this loss of acinar tissue, one would expect a very high density of neuronal staining in the regions between islets in the CF pancreas. Instead, we observe a paucity of pan-neuronal TUJ1 immunostaining in the non-endocrine portions of the CF pancreas in humans, suggesting a marked loss of pancreatic innervation. In ferrets, the picture differs in the endocrine CF pancreas. Although there were numerous TUJ1 immunostaining structures within and immediately surrounding ferret CF islets, these structures exhibited a thicker shape and were abnormally distributed compared with CON. In terms of parasympathetic neuronal markers, we observed very few VAChT-staining neuronal structures in both human and ferret CF pancreata, indicating that CF induces loss of parasympathetic innervation. In terms of sympathetic neurons, tyrosine hydroxylase immunostaining axonal structures were present in CON human pancreas but to a lesser degree in the human CF pancreas. However, we were only able to examine one CF human pancreas. Given the lack of a specific functional readout for sympathetic action on islets, we feel that conclusions would be premature at this time. Firmer conclusions are possible in ferrets, where there was an extensive network of tyrosine hydroxylase-positive axons throughout the islets of CON pancreata. Although tyrosine hydroxylase-positive cell bodies were observed in CF islets, there were no tyrosine hydroxylase-positive axons observed, possibly indicating loss of this sympathetic neuronal plexus.

Other forms of neuronal dysfunction have been observed in CF. CFTR is expressed in various locations in the nervous system, including in neurons of visceral autonomic ganglia [41]. CFTR is also expressed in Schwann cells, where its loss may contribute to neuronal dysfunction in CF [42]. CF mice have diminished expression of the parasympathetic marker vasoactive intestinal peptide in axons in the pancreas [43] and other tissues impacted by CF, including lungs. However, it is not known whether PP secretion is impaired in CF mice. Because CF mice do not develop substantive exocrine pancreatic disease, this suggests that the loss of vasoactive intestinal peptide-expressing parasympathetic neurons in CF disease may be independent of exocrine pancreatic disease. Our data suggest that neuronal loss in CF occurs early in life and could be compatible with a variety of mechanisms, including collateral disruption during exocrine inflammation and/or CFTR-dependent mechanisms related to Schwann cells or loss of neuronal CFTR expression. The CF ferret, which develops spontaneous diabetes unlike the CF mouse, could serve as an ideal model to test how parasympathetic innervation loss might contribute to diabetes in CF.

The physiological secretion of PP is dependent upon parasympathetic innervation, via the vagus nerve [23]. Consumption of nutrients and hypoglycemia each independently raise parasympathetic efferent tone in the vagus nerve in a manner that induces PP secretion from islets. Consistent with the dependence of PP secretion on parasympathetic innervation, vagotomy greatly impairs PP secretion in response to physiological stimuli [44]. Likewise, diabetic autonomic neuropathy also impairs PP secretion [44]. This illuminates an advantage of studying very young children and ferrets with CF at an age before the possibility of hyperglycemia-related neuropathy. We found that PP secretion in response to OGTT is already impaired by a very young age, both in ferrets and humans. Interestingly, these low PP levels were not due to an absence of PP cells, as these were abundant in the islets of the CF pancreas, both in humans and ferrets. Consistent with this, we observed intact secretion of PP in response to MMTTs in CF ferrets, though this differs from humans with CF whose PP levels do not respond to mixed meal testing [45]. The finding of intact PP cells and responsiveness to mixed meal testing in CF ferret pancreas is not surprising, as we previously observed relative increases in PP mRNA starting at phase II and also glucagon mRNA starting in phase III [25], and increased abundance of PP cells in the CF pancreas has previously been described for PwCF [16]. Importantly, we also found that isolated CF ferret islets are capable of secreting PP when perfused with arginine, though our data cannot rule out a partial islet-autonomous loss of PP secretory capacity. Had arginine failed to stimulate PP from CF islets, then this would have suggested the possibility of a completely different mechanistic origin of low PP in CF involving PP cell dysfunction. Taken together, these findings indicate that reduced PP secretion is likely not solely due to a cell-intrinsic defect in the PP cells but rather results from a failure of functional parasympathetic innervation to islets initiated by CF very early in life.

Loss of pancreatic innervation has implications beyond PP secretion. Insulin secretion is enhanced by parasympathetic tone, particularly early-phase secretion [23]. Impaired early-phase insulin secretion is well described in PwCF, even in those without detectable glycemic abnormalities [3,4]. Likewise, we have observed deficient early-phase insulin secretion in CF ferrets and CF ferret islets [21,22]. Given the deficiency of parasympathetic VAChT staining in the CF pancreas, it is possible that failed parasympathetic innervation of islets contributes to this poor early-phase insulin secretion in CF. Glucagon secretion in response to hypoglycemia is thought to be augmented by sympathetic and potentially parasympathetic innervation [23]. Hypoglycemia-induced glucagon secretion is impaired in PwCF [5], and we find this impairment in CF ferrets as well. Similarly to PP cells, increased abundance of alpha cells in the CF pancreas has previously been described for humans [16] and ferrets [25]. This indicates that it is not a lack of alpha cell mass that is responsible for poor glucagon secretion and implies that failed sympathetic and/or parasympathetic innervation may contribute to the deficiency of glucagon secretion in response to physiological stimuli in CF.

It is possible that the early life pancreas is uniquely susceptible to permanent damage to its neuronal innervation. Our data indicate that the loss of general innervation in the CF ferret pancreas occurs in the first two months of life, sometime between the newborn period and phase III. Interestingly, acute and chronic pancreatitis with onset in adulthood often does not reduce PP secretion, even in response to food, even when the pancreatitis has been severe enough to cause diabetes mellitus [46–48], though reduced PP levels have been observed in some CP populations, especially those with diabetes [48–50]. Nonetheless, neuronal density in the pancreas is increased in adults with chronic pancreatitis [51]. Likewise, when we delayed the onset of exocrine pancreatic disease until after 30 days of age by use of VX-770 in CFTR-G551D ferrets, substantial neuronal innervation of the pancreas was observed. In fact, the density of TUJ1 staining neurons was increased in the pancreases of CF_delay_ ferrets. This finding could simply be a consequence of the dramatic loss of acinar tissue in the CF_delay_ pancreas, such that axons become more densely packed as the acinar cells involute (i.e., same number of neurons in a much smaller volume). Alternatively, there could be excess generation of axons and nerves in the CF_delay_ pancreas. Our data are unable to distinguish between these possibilities. Consistent with the susceptibility of the parasympathetic system to early life perturbations, VAChT staining in the CF newborn ferret pancreas is disordered even before the onset of overt exocrine pancreatic disease.

The findings of reduced neuronal innervation of the pancreas in CF may also help explain why PwCF typically experience little pancreatic pain despite extensive exocrine pancreatic disease [52]. By contrast, persons with chronic pancreatitis exhibit increased neural density [51], which, combined with neural remodeling and neuritis, is thought to contribute to the extensive pain experienced in this condition [53].

A large portion of beta-cells in ferrets with CF co-expressed insulin and tyrosine hydroxylase. Relevantly, the tyrosine hydroxylase gene is nearly adjacent to the insulin gene, both in humans [54] and in ferrets (NCBI reference sequence: NW_025421288). Early during beta-cell differentiation, tyrosine hydroxylase is expressed, sharing open chromatin with insulin. As beta-cells become fully differentiated, selective DNA methylation silences tyrosine hydroxylase but not insulin [54]. Furthermore, beta-cell stressors can result in loss of this methylation and re-expression of tyrosine hydroxylase, contributing to dopamine synthesis, which in turn reduces insulin secretion by beta-cells [37]. Thus, tyrosine hydroxylase expression by beta-cells in the CF ferret pancreas may represent another mechanism that impairs insulin secretion and contributes to CFRD in ferrets. The one human CF pancreas we examined did not exhibit discernable tyrosine hydroxylase staining in insulin-positive cells.

Strengths of our study include that we examine both human and ferret pancreas samples, allowing interspecies comparisons. Importantly, both ferrets and humans experience the same islet hormonal disturbances, namely (i) impaired early insulin secretion in response to hyperglycemia, (ii) impaired glucagon secretion in response to hypoglycemia, and (iii) impaired PP secretion in response to feeding and hypoglycemia. There were similarities in our neuronal marker studies across the two species in that there is a loss of neuronal innervation of the CF pancreas, especially parasympathetic nerves. It is important to bear in mind that we uncovered differences in the neuronal patterns in the pancreas of ferrets versus humans, and thus ferrets may not serve as a perfect model of CF-induced neuronal disruption. Other limitations of our study include that quantitative morphometrics were not possible with the limited pancreatic samples available and due to challenges in complete systematic sampling of the CF pancreas. Advanced negative controls, such as antigen knockout or blocking peptides, were not available for our studies. Furthermore, microscopic studies cannot definitively inform whether a given tissue or cell type is functionally innervated or denervated. Ideally, we would have performed functional studies such as vagal stimulation to more definitively determine whether there was a denervation of the pancreas. However, owing to the complexities of CF ferret husbandry, these studies could not be performed. Our studies were performed exclusively under long light conditions, which are required for ferret fertility. It is possible that shorter light cycles could influence the propensity of ferrets to experience hormonal and metabolic disturbance. Another limitation of our study was that in many cases it was not possible to obtain age-matched pancreatic tissue samples, leading to comparison of samples from different ages and stages of pancreatic growth and maturation. Our functional data, using PP secretion as a marker of parasympathetic innervation, suggest that defects exist and persist from early in childhood both in humans and in ferrets.

In summary, we find that the CF pancreas is impacted by marked loss and remodeling of neuronal innervation. The qualitative aspects of the neuronal changes are correlated with quantitative impairments in PP secretion, glucagon secretion, and early-phase insulin secretion in response to physiologic stimuli mediated by the CNS. These findings imply that denervation has functional consequences for endocrine hormone secretion in CF.

## Clinical perspectives

- Persons with CF have impaired pancreatic peptide secretion, delayed insulin secretion, dysregulated glucagon secretion, and often a paucity of pancreatic pain. Because pancreatic innervation is involved in these processes, we examined a marker of pancreatic parasympathetic action and neuronal markers in humans with CF and in an animal model of CF pancreatic disease.
- Young children and ferrets with CF exhibited impaired secretion of PP, indicating failed pancreatic islet parasympathetic action. Pancreata from humans and ferrets with CF exhibited altered and/or diminished staining of general, parasympathetic, and sympathetic nervous system markers. Delaying the onset of pancreatic disease in ferrets restored neuronal markers and oral glucose-induced PP secretion.
- These data are consistent with CF inducing early life impairments in pancreatic neuronal innervation. These deficits may contribute to pancreatic dysfunction.

## Supplementary Material

Supplementary Figures S1-S16 and Supplementary Table S1

Supplementary Video S1

Supplementary Video S2

## Data Availability

Individual ferret data are reported in the Supplementary Data file. Human data can not be publicly posted owing to consent restrictions. Image files and human data are available upon reasonable academic request and regulatory approval when necessary.

## Open Access

Open access for this article was enabled by the participation of the University of Iowa in an all-inclusive Read & Publish agreement with Portland Press and the Biochemical Society under an agreement with the Big Ten Alliance.

## Abbreviations

CF: cystic fibrosis
CON: control
CFRD: CF-related diabetes
CFTR: CF transmembrane conductance regulator
CNS: central nervous system
MMTTs: mixed meal tolerance tests
NGS: normal goat serum
OGTT: oral glucose tolerance testing
PBS: phosphate-buffered saline
PP: pancreatic polypeptide
PwCF: people with cystic fibrosis
VAChT: vesicular acetylcholine transporter
WT: wild-type
