# Supplementary material for: Structural and functional impact of cystic fibrosis on endocrine pancreatic innervation
Source: Clin Sci (Lond). 2026 Sep 29;140(10):2179–97. doi: 10.1042/CS20260182 (PMC13624406; doi:10.1042/CS20260182)
Supplement: Supplementary file 1 [file CS-2026-0182_suppl.pdf]

SUPPLEMENTAL FIGURES: Figure S1

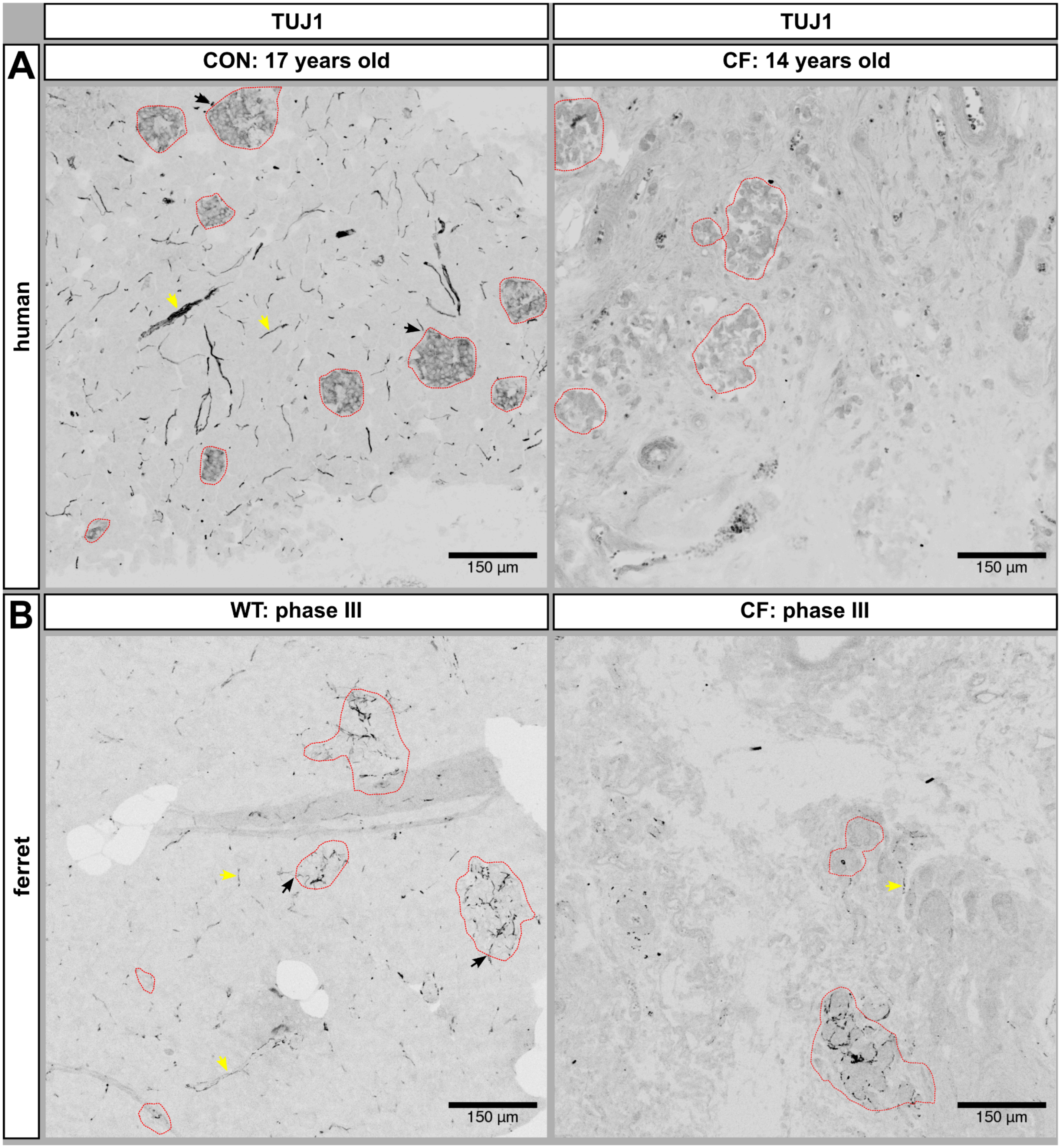

Figure S1: Pan-neuronal pancreatic innervation. Class III beta-tubulin (TUJ1 channel, black) staining of human (A) and ferret (B) pancreas on 10 µm paraffin sections. Same micrographs as figure 1. Greyscale has background purposely elevated to allow full examination. Left column: control human (CON), wildtype ferret (WT); right column CF. Ages (human) or pancreatic stages (ferret) are indicated. Human micrographs representative of 3 independent images. Ferret micrographs representative of over 10 images collected from 2 animals. Beta-islets are circled with red dotted lines, though small and irregular islets are not marked. Examples of TUJ1+ axonal processes are shown with yellow arrows. Examples of TUJ+ axons adjacent or interfacing with islets are highlighted with white arrows. These are from the same images as shown in Figure 1.

Figure S2

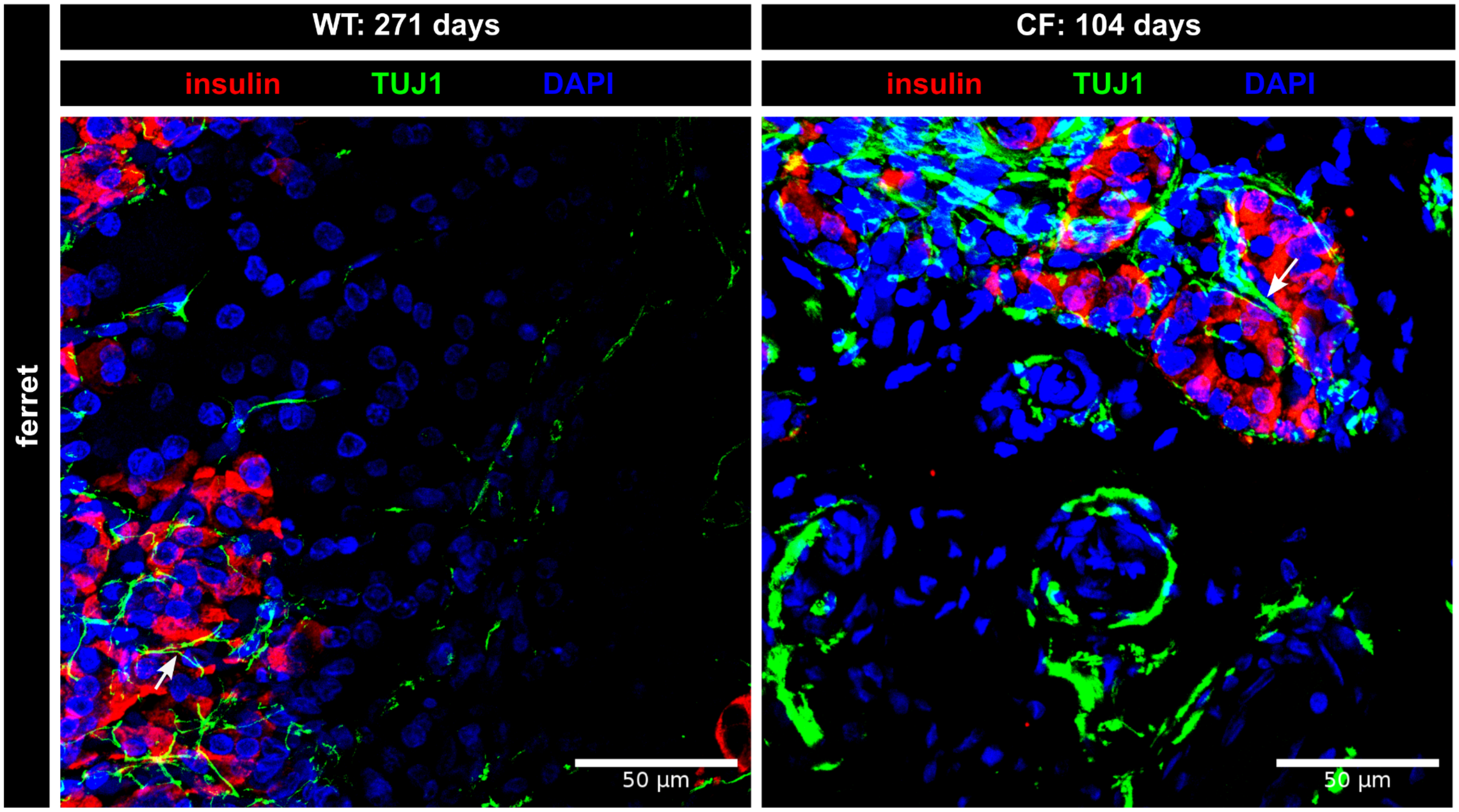

Figure S2: Pan-neuronal innervation in ferret pancreas at higher magnification. Class III beta-tubulin (TUJ1, green) and insulin (red) co-staining of ferret pancreas on 10 µm paraffin sections. Blue is DAPI. Left column: wildtype ferret (WT); right column CF. Ages are indicated in the figure. Micrographs representative of over 10 images collected from 2 animals. An example of a normal-thickness intraislet axon is highlighted with a white arrow in the left panel, whereas a thicker intraislet axon is highlighted in the right panel.

Figure S3

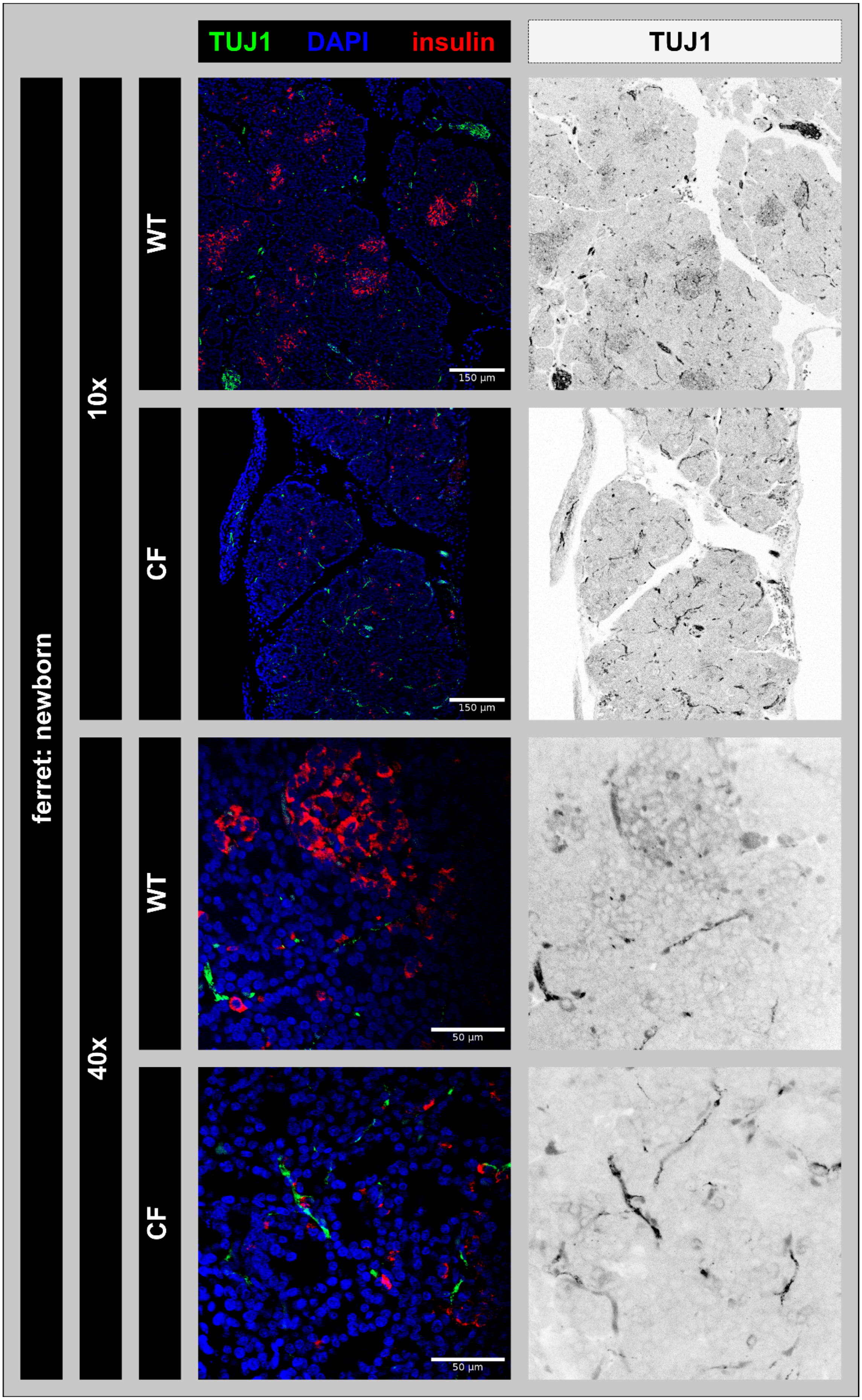

Figure S3: Pan-neuronal pancreatic innervation in newborn ferrets. Immunostaining of newborn ferret pancreas on 5 µm paraffin sections. Left column - full color: tubulin B3 (TUJ1, green) and insulin (red). Blue is DAPI. Right column - TUJ1 channel only in black. Greyscale has background purposely elevated to allow full examination. Top two rows, lower magnification. Bottom two rows - higher magnification. Genotypes indicated in each row. Micrograph representative of 7 images per genotype.

Figure S4

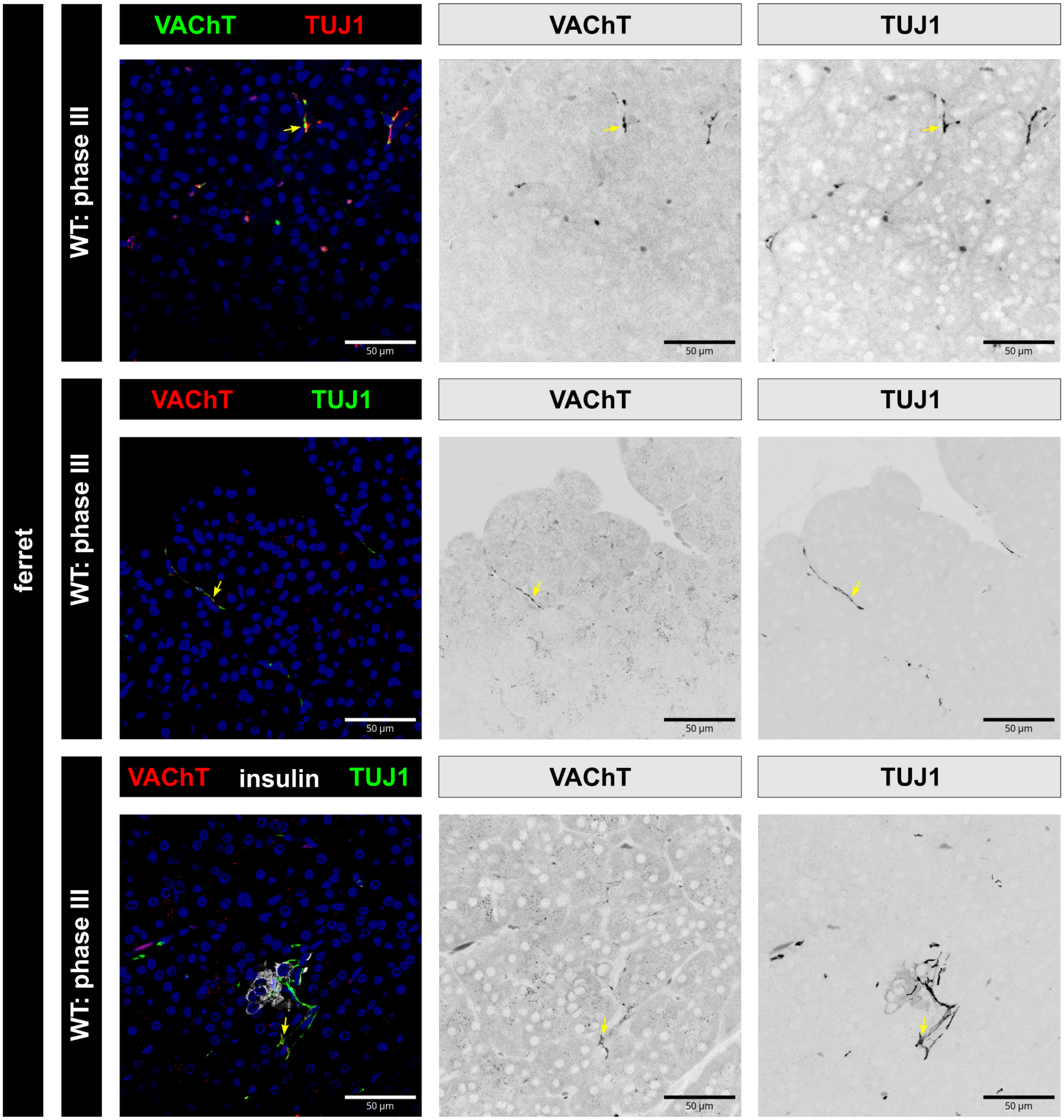

Figure S4: Co-localization of vesicular acetylcholine transferase (VAChT) and Class III beta-tubulin (TUJ1) in wildtype (WT) ferret pancreas. Each row is a different pancreatic sample, all from phase III WT ferrets. In row 1, VAChT utilized a green secondary antibody and TUJ1 a red antibody. This was reversed in rows 2 and 3, with VAChT being red and TUJ1 green. Row 3 added insulin immunostaining in the far-red, shown as white. The left-most column shows all colors, including DAPI as blue. The middle column shows the VAChT channel only; the right column, TUJ1 channel only. Greyscale has background purposely elevated to allow full examination. Yellow arrows point to example VAChT+ varicosities within a TUJ1+ axonal processes.

Figure S5

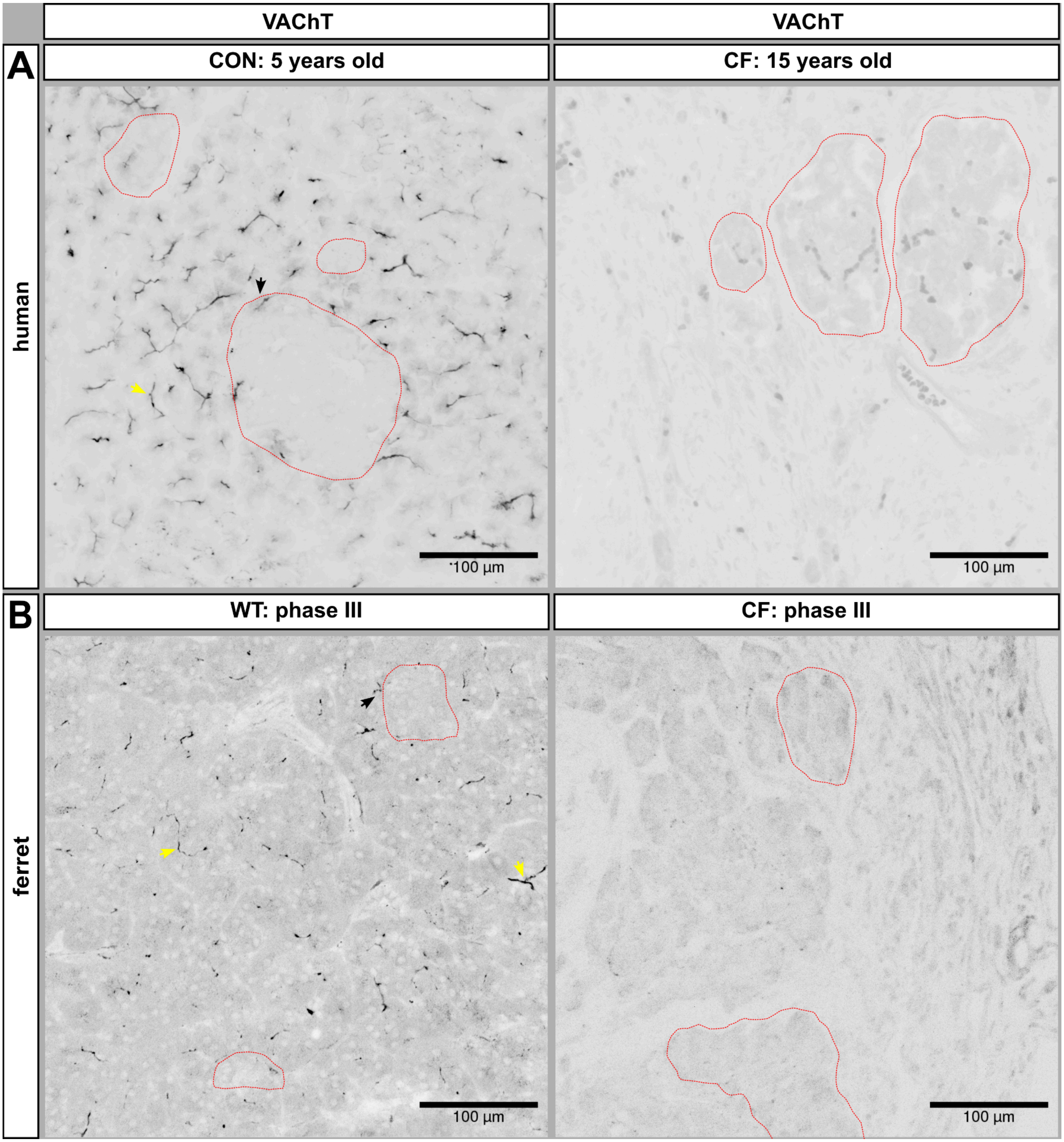

Figure S5: Pancreatic parasympathetic innervation. Vesicular acetylcholine transporter (VAChT channel, black) in human (A) and ferret (B) pancreas on 10 µm paraffin sections. These correspond exactly to the micrographs in Figure 2. Top row: human samples. Bottom row: ferret. Left columns: control human (CON), wildtype ferret (WT); right columns: CF. Ages are indicated in the figure. Greyscale has background purposely elevated to allow full examination. For example, autofluorescence in red blood cells is evident in the human CF micrograph. Beta-islets are circled with red dotted lines, though small and irregular islets are not marked. Examples of VAChT+ axonal processes are shown with yellow arrows. Examples of VAChT+ axons adjacent or interfacing with islets are highlighted with black arrows. These are from the same images as FIgure 2.

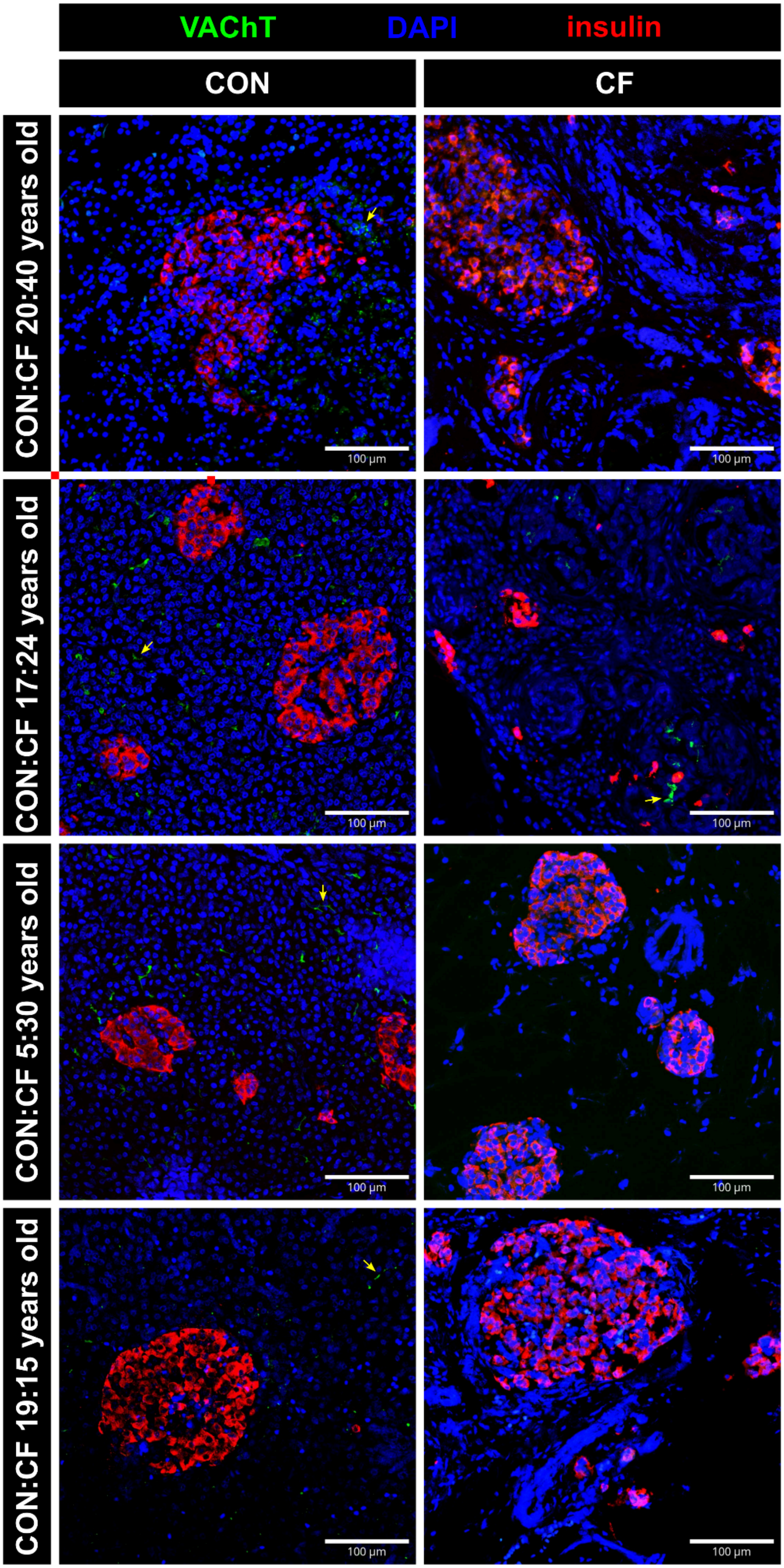

Figure S6: Pancreatic parasympathetic innervation in human pancreas. Samples are from humans without CF (controls, CON) and with CF (CF) as indicated. Vesicular acetylcholine transferase (VAChT, green) and insulin (red) co-staining of 10 µm paraffin sections. Blue is DAPI. The CON:CF dyads are each paired by staining / imaging date. Micrographs representative of 2-6 images per pancreas. The 5 year old control pancreas is the same as shown in Figure 2; likewise for the 15 year old CF pancreas. An example of neuronal VAChT staining, when present, is highlight by a yellow arrow per panel.

Figure S7

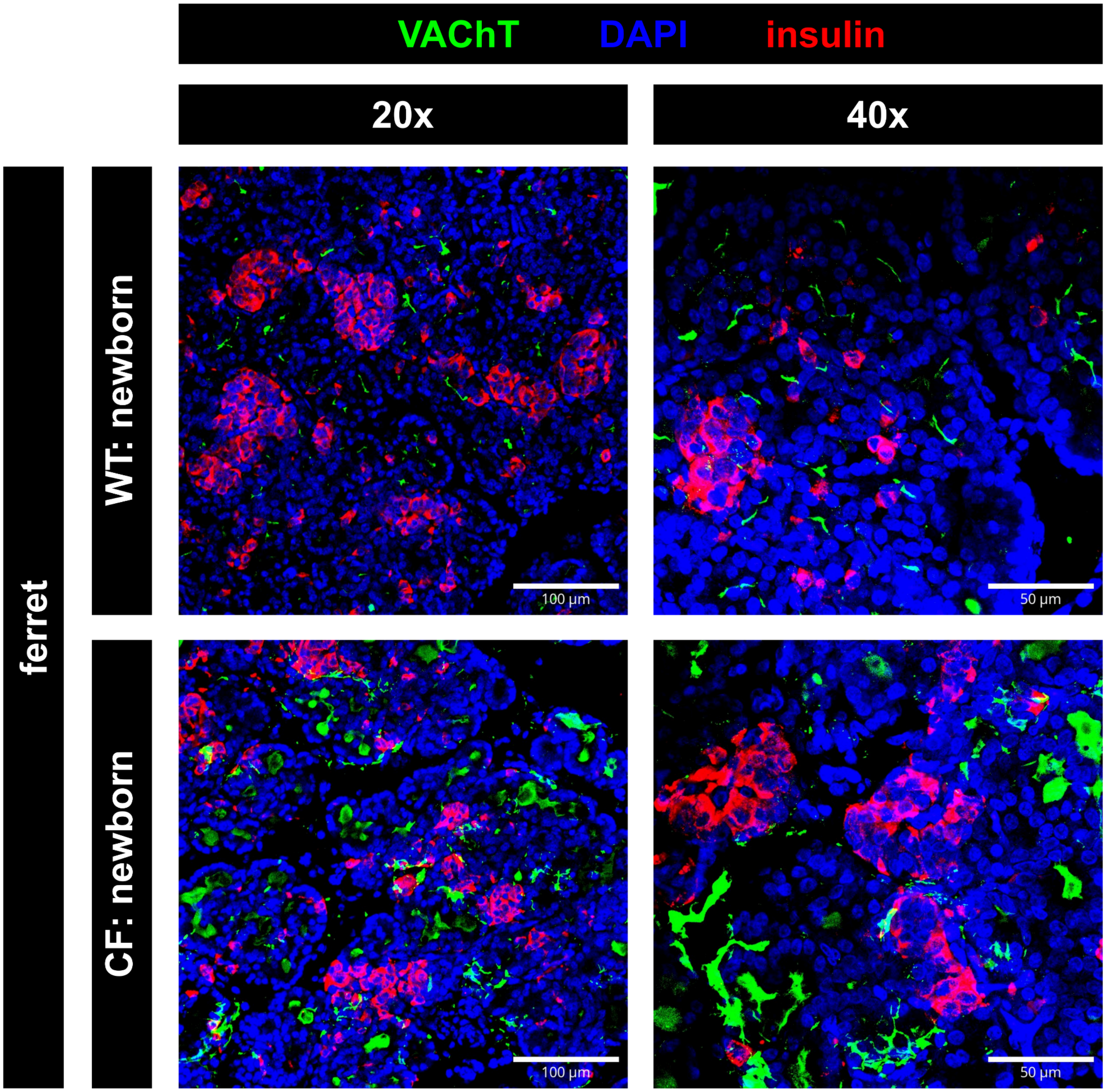

Figure S7: Pancreatic parasympathetic innervation in newborn ferrets. Vesicular acetylcholine transferase (VAChT, green) and insulin (red) co-staining of ferret pancreas on 10 µm paraffin sections. Blue is DAPI. Top rows WT; bottom rows CF. Images representative of over 10 images per genotype.

Figure S8

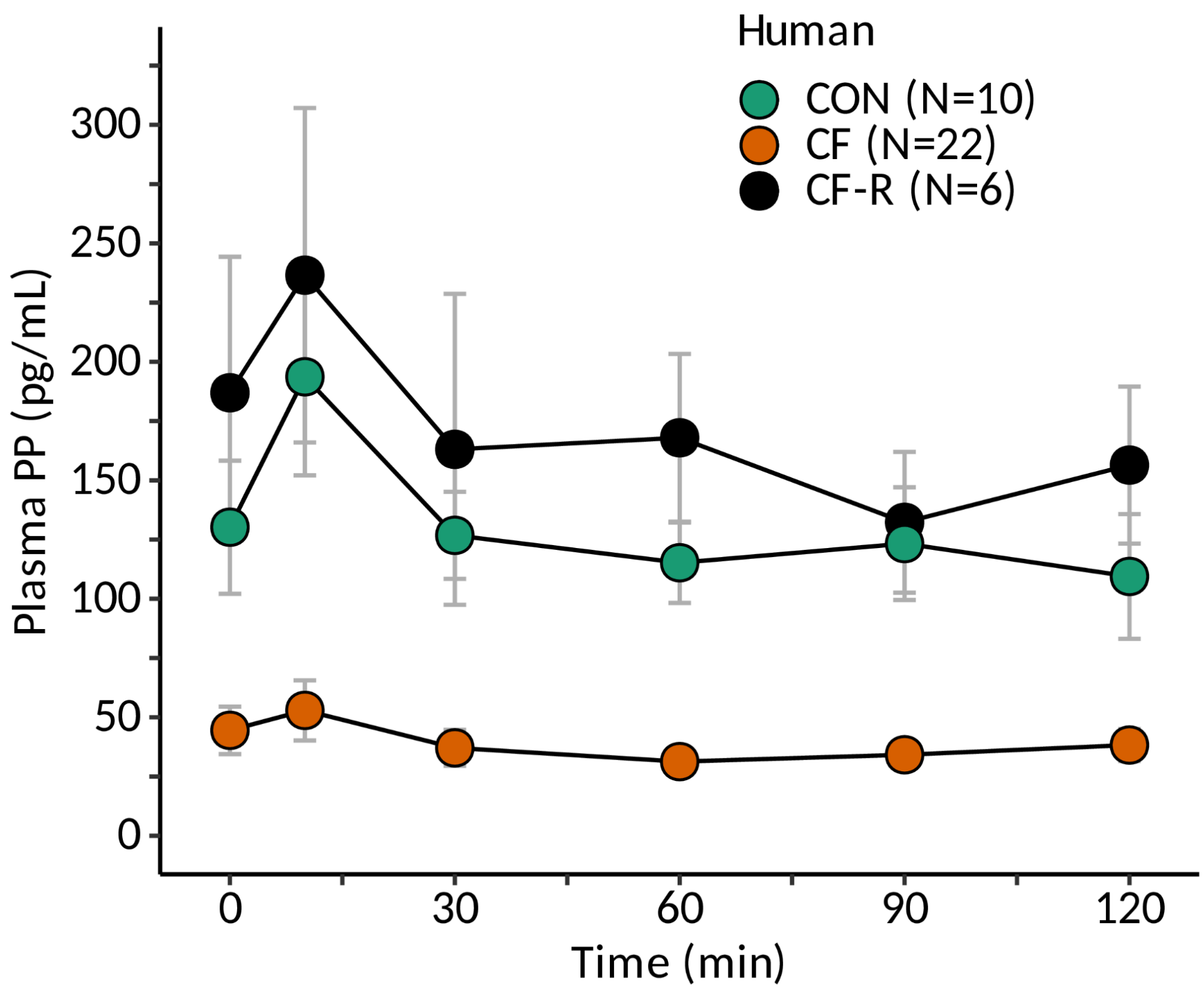

Figure S8: PP levels in response to oral glucose tolerance testing in humans. Data shown includes controls (CON), CF with mutations that do not retain CFTR function (CF), and third group with cystic fibrosis but with at least one mutation that retains residual CFTR function (CF-R).

Figure S9

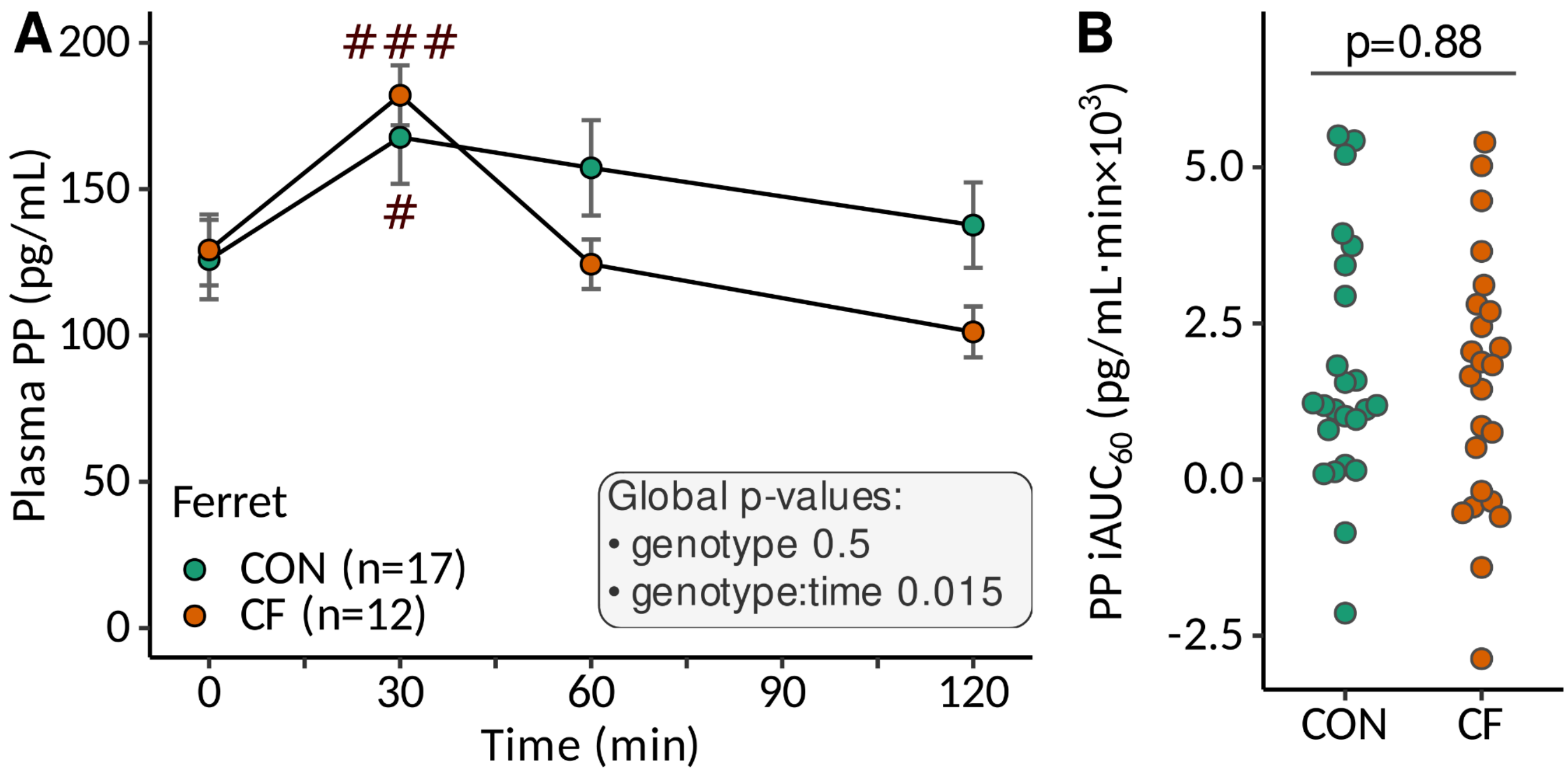

Figure S9: PP levels in response to mixed meal tolerance testing. Serum PP levels in ferrets (**A-B**). Time course (**A**) and incremental AUC for the first 60 minutes (**B**, $iAUC_{60}$) are shown. Several ferrets underwent repeated testing, and thus N represents the number of ferrets rather than the number of tests examined. (**A**) Significance was determined by mixed effects modeling with the arising global p-values related to genotype details in insets. Posthoc p-values are indicated by # p<0.05, ### p<0.0005 for difference from baseline for that specific genotype. Posthoc testing revealed no differences between genotype for any specific timepoint. (**B**) P-value was determined by Wilcoxon Wilcoxon rank sum test, averaging iAUC60 values for repeated tests.

Figure S10

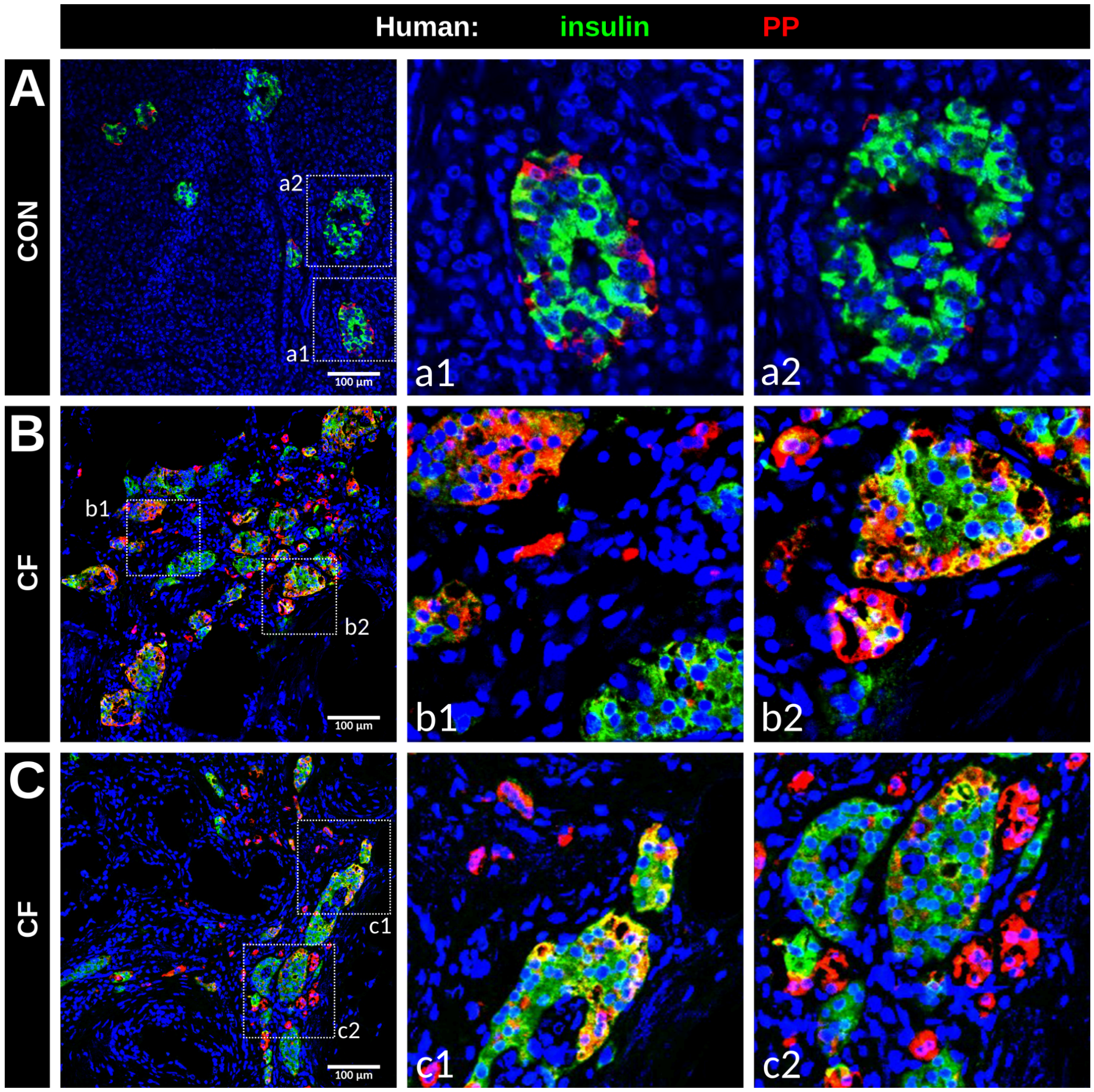

Figure S10: Immunostaining of PP- and beta-cells in human pancreata. Each row represents a different individual without (**A**, control "CON") or with CF (**B,C**). Green: insulin. Red: pancreatic polypeptide. Blue: DAPI. Middle and right columns show the regions indicated in the left column. Human CF micrographs representative of images collected from 5 separate CF pancreata.

Figure S11

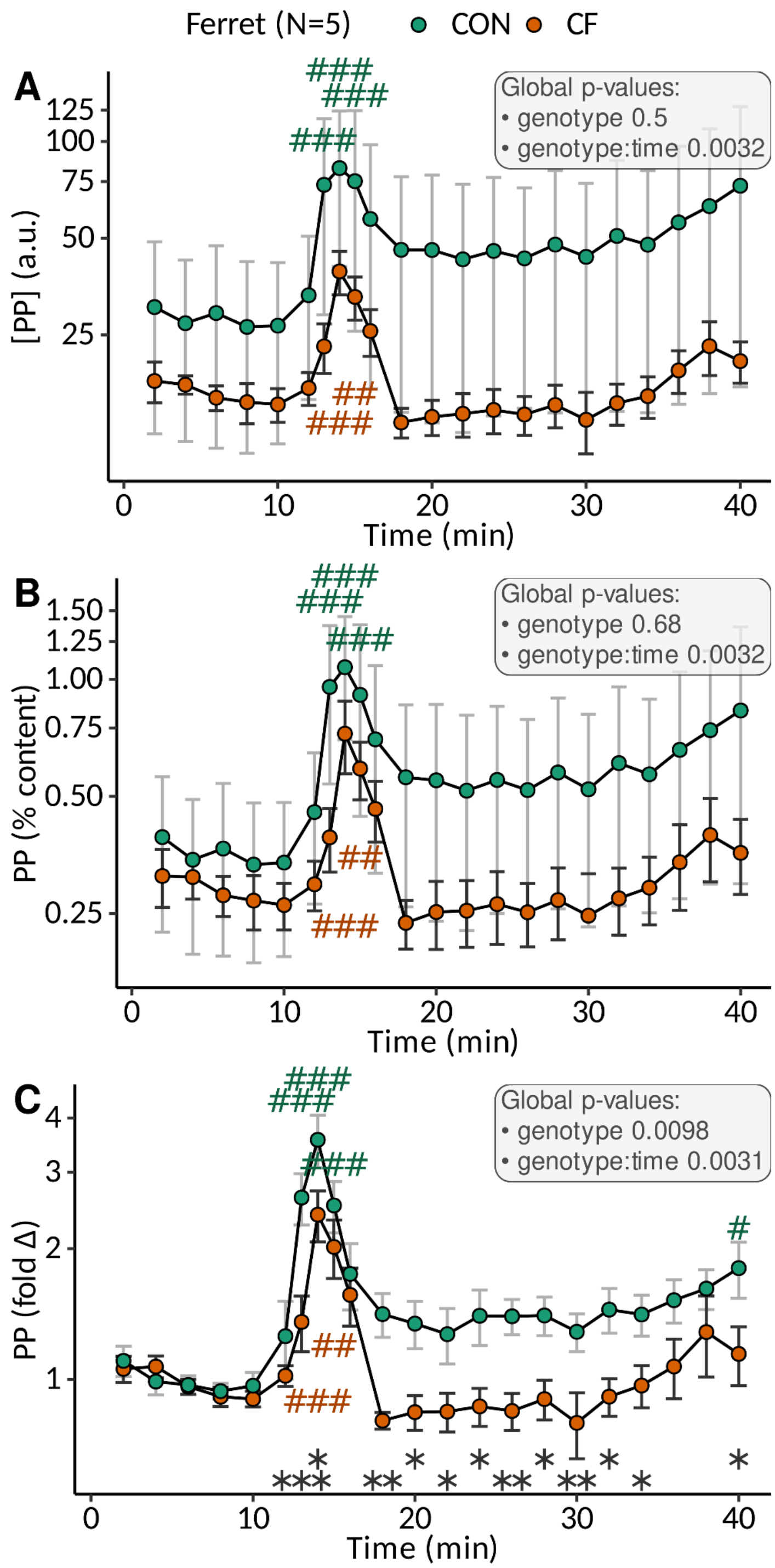

Figure S11: Perifusion of islets. Arginine was introduced into the perifusate at 10 minutes for islets from CON (green) and CF (orange) ferrets. (**A**) Perifusate PP concentration. (**B**) Fractional secretion compared to post-perifusion islet PP content. (**C**) Fold change compared to the mean 0-8 minute baseline. Significance was determined by mixed effects modeling on log-transformed data with the arising global p-values related to genotype found in the insets. When indicated by global p-values, posthoc p-values were calculated and are indicated by * p<0.05, ** p<0.005,, *** p<0.0005 for difference between CF and CON at the specified time points, and # p<0.05, ## p<0.005, ### p<0.0005 for difference from baseline for that specific genotype, as color-coded. Islet PP content did differ between groups (CON 7,518 ± 1,806 pg/islet equivalent; CF 5,943±734; p=1.0).

Figure S12

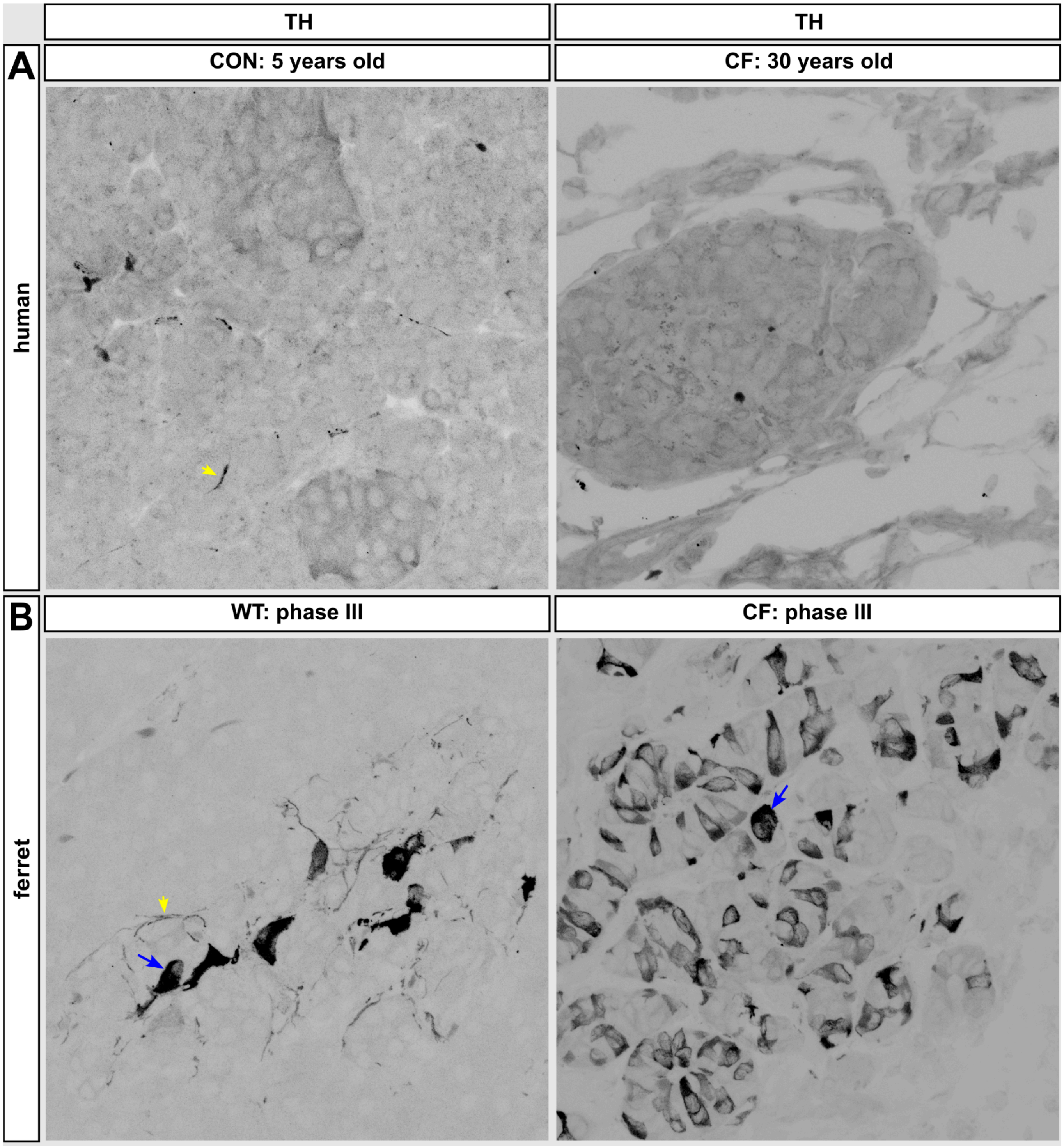

Figure S12: Tyrosine hydroxylase (TH) immunostaining. Samples were from human (**A**) and ferret (**B**) pancreas. Micrographs same as Figure 8, but only showing the TH channel as black. Top rows: human; bottom rows: ferret. Left columns: control (CON, human) or wildtype (WT, ferret); right columns: CF. Ages are indicated in the figure. Human micrographs representative of 3 independent images from 1 pancreas. Ferret micrographs representative of images from 3 WT and 2 CF pancreata, each undergoing at least 6 independent images. Greyscale has background purposely elevated to allow full examination. Yellow arrows indicate example TH+ axons. Blue arrows indicate example TH+ insulin+ cells. These are from the same images as shown in FIgure 8.

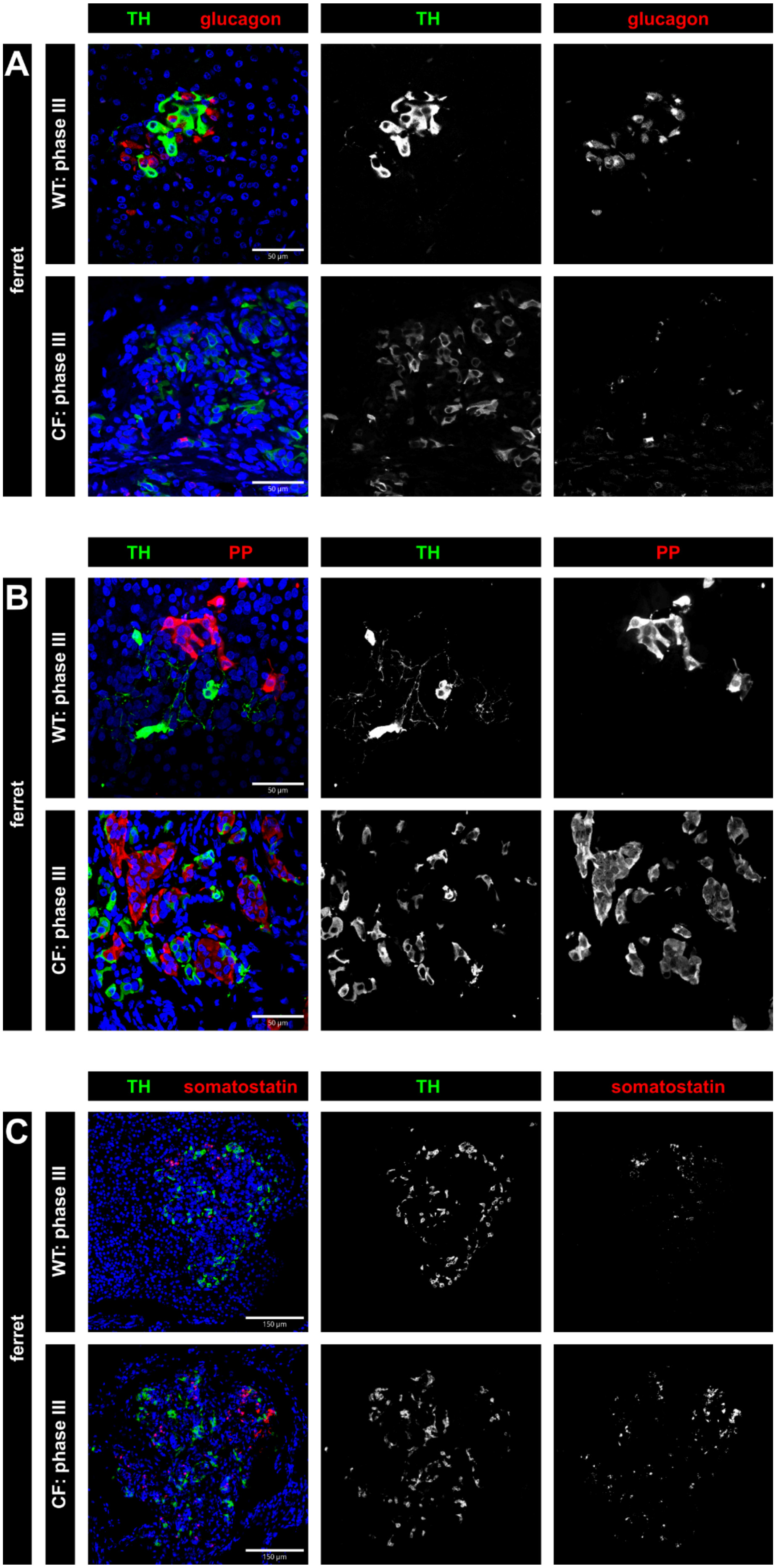

Figure S13: Ferret pancreas tyrosine hydroxylase (TH) expression does not co-localize with glucagon, pancreatic polypeptide (PP) or somatostatin. Green=TH, red=hormone, blue=DAPI. (**A**) Glucagon detected by immunofluorescence in 10-micron paraffin sections. Single confocal slice shown to minimize artifactual color overlap. (**B**) PP detected by immunofluorescence in 10-micron paraffin sections. Maximum intensity projections shown. (**C**) SST detected by FISH in 4-micron paraffin sections. Micrographs representative of 4-6 images per genotype. Top rows wildtype (WT); bottom rows CF.

Figure S14

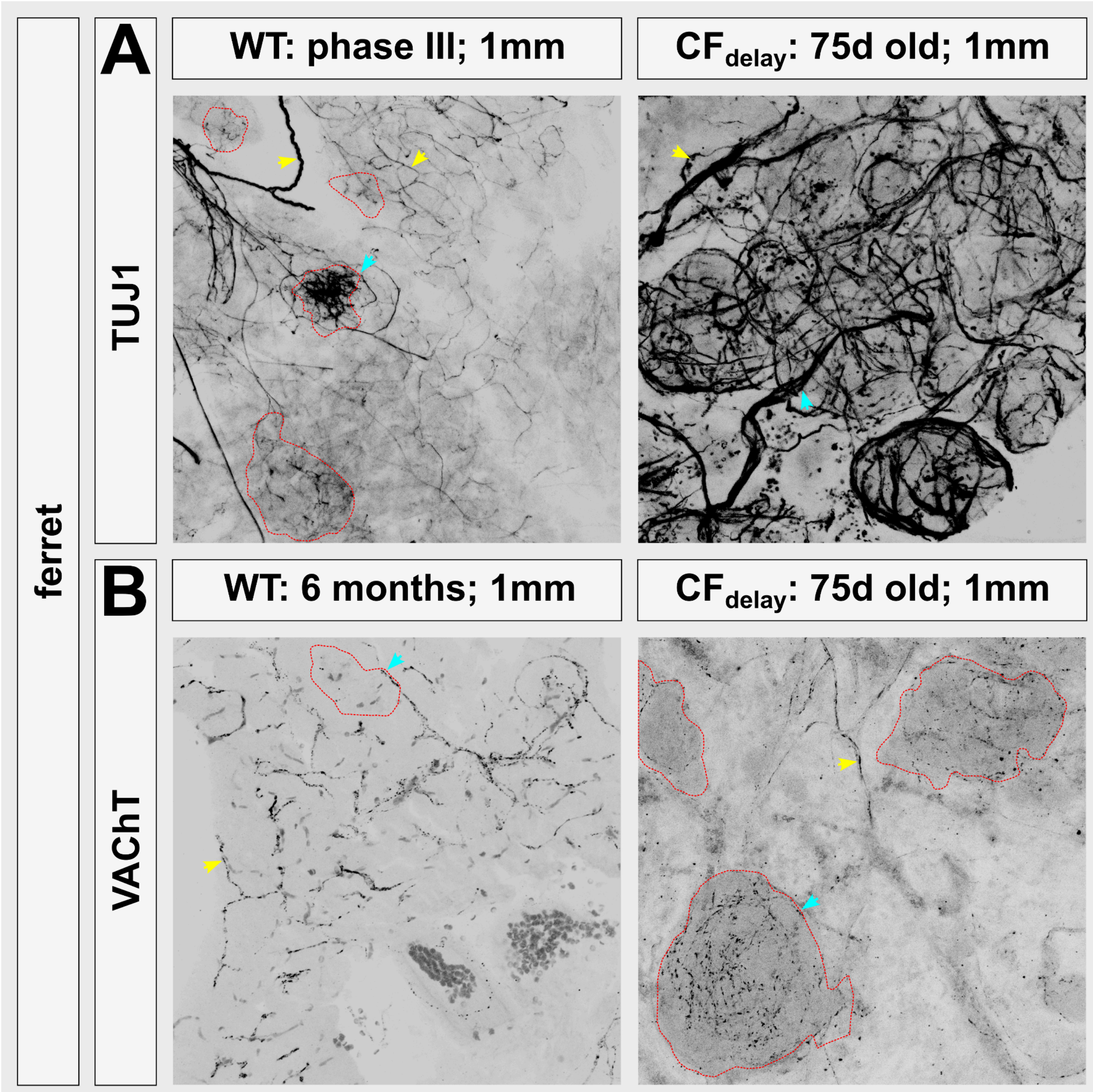

Figure S14: Delay of CF disease spares subsequent pancreatic neuronal innervation. CF_{delay} ferrets harboring *CFTR*^{G551D} were maintained on VX770 until 30 days old. CF_{delay} pancreata were sampled at 75 days of age. Wildtype (WT, left column) and CF_{delay} ferrets (right column). (**A**) Top row, pan-neuronal marker TUJ1 in 1 mm whole-mount pancreas. Black=TUJ1. (**B**) Bottom row, VAChT immunostaining on 1mm whole-mount pancreas. Black=VAChT. WT micrographs representative of at least 5 independent images; CF_{delay} micrographs representative of images from 5 pancreata. Images are the same micrographs as in Figure 9, but examining only the indicated fluorescent channel. Greyscale has background purposely elevated to allow full examination. Beta-islets are circled with red dotted lines, though small and irregular islets are not marked nor are islets in top right as islets occupy most of the figure. Examples of TUJ1+ axonal processes are shown with yellow arrows. Examples of TUJ+ axons adjacent or interfacing with islets are highlighted with teal arrows.

Figure S15

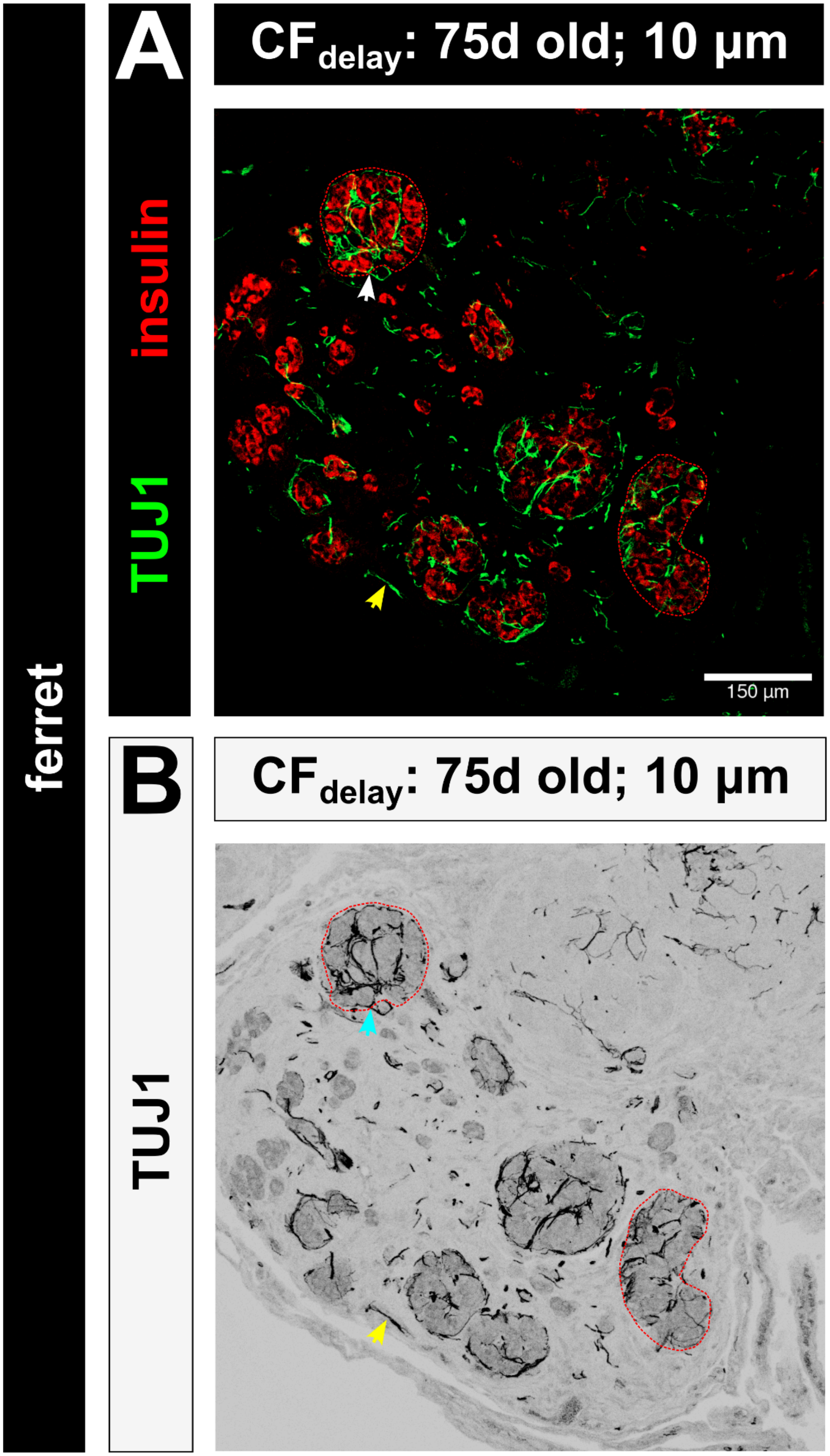

Figure S15: Delay of CF disease spares subsequent pancreatic TUJ1+ neuronal innervation, as examined in thin sections. CF$_{delay}$ ferrets harboring *CFTR^{G551D}* were maintained on VX770 until 30 days old. CF$_{delay}$ pancreata were sampled at 75 days of age. Only CF$_{delay}$ ferrets are shown, as similar images from WT ferrets are shown in earlier figures. (**A**) Red=insulin, Green = TUJ1. (**B**) The same image showing only the TUJ1 channel in greyscale. Greyscale has background purposely elevated to allow full examination. Selected beta-islets are circled with red dotted lines. Examples of TUJ1+ axonal processes are shown with yellow arrows. Examples of TUJ+ axons adjacent or interfacing with islets are highlighted with white (A) or teal (B) arrows.

Figure S16

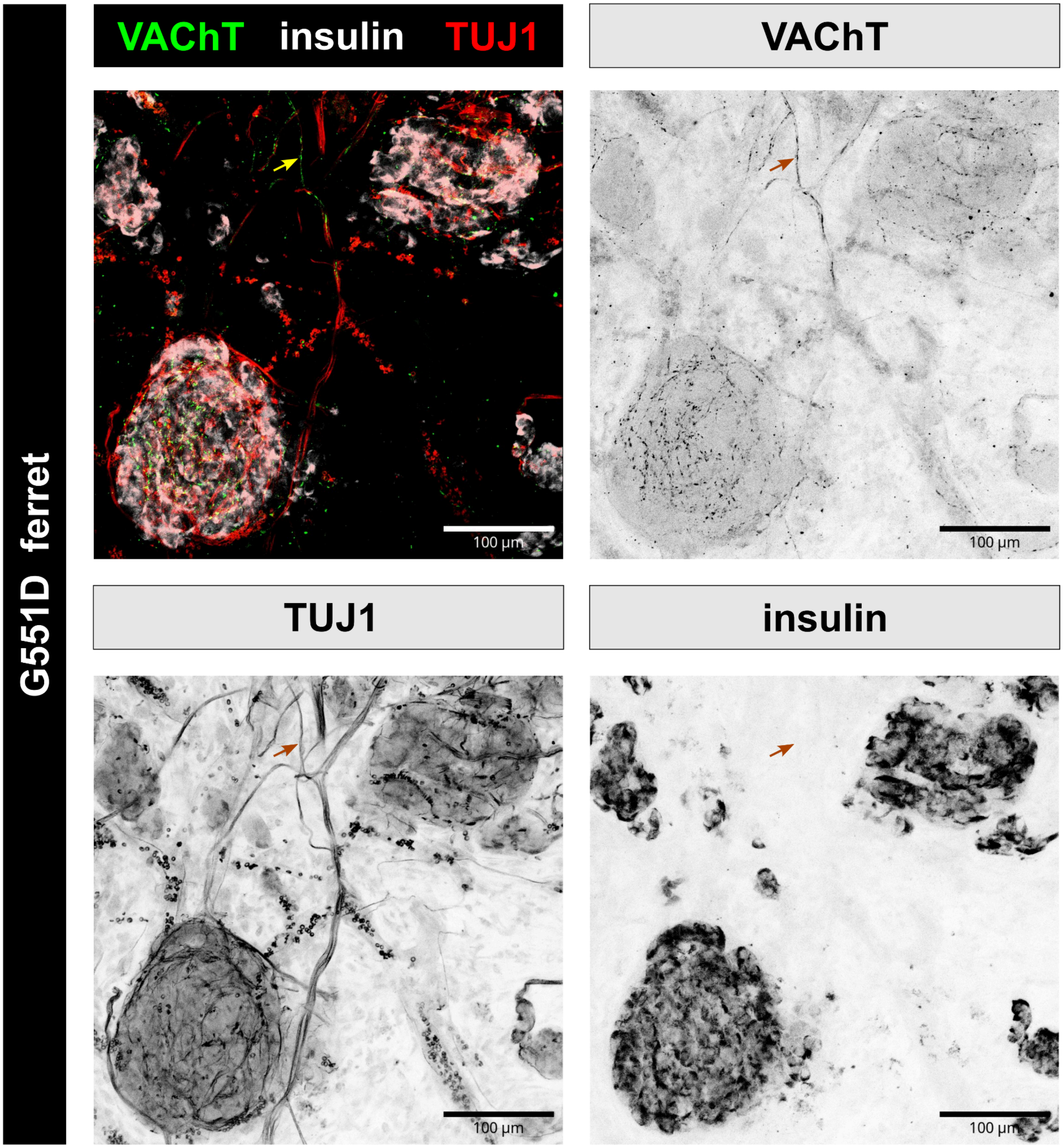

Figure S16: Co-localization of vesicular acetylcholine transferase (VAChT) and Class III beta-tubulin (TUJ1) in wildtype (WT) ferret pancreas. Pancreas from a 75 day old CF_{delay} ferret harboring *CFTR*^{G551D} who was maintained on VX770 until 30 days old. Top left: VAChT green, TUJ1 red, insulin white. Red=insulin. Wildtype (WT, left column) and CF_{delay} ferrets (right column). (**A**) Top row, pan-neuronal marker TUJ1 in 1 mm whole-mount pancreas. Green=TUJ1. (**B**) Bottom row, VAChT immunostaining on 1mm whole-mount pancreas. Top right: VAChT channel in grey scale. Bottom left: TUJ1 channel in grey scale, note bright RBC. Bottom right: insulin channel in greyscale. Top Left: Yellow arrow point to example VAChT+ varicosity within a TUJ1+ axonal process. Other panels: arrow is orange.

SUPPLEMENTAL VIDEOS - Legend

Video S1 and S2: Rendering of 1 mm thick immunofluorescence ferret pancreas micrographs, rotating 30 degrees in either direction to allow three-dimensional visualization. **S1** is pancreas from wildtype ferret. **S2** is pancreas from CF_{delay} ferret. Colors: Green = TUJ1, Red = insulin, blue = DAPI. Note that in the CF_{delay} image, the blue DAPI signal is very weak.

SUPPLEMENTAL TABLE

Table S1: Antibodies.

**Primary AB**	**Host**	**Maker**	**Cat #**	**RRID**
Glucagon	rabbit	Cell Signaling	2760	AB_659831
Insulin	guinea pig	Agilent	A0564	AB_10013624
Pancreatic polypeptide	rabbit	Millipore	AB939-I	AB_92383
Tubulin β 3 (TUJ1 clone)	mouse	Biolegend	801202	AB_2313773
Tyrosine Hydroxylase	mouse	Immunostar	22941	AB_572268
VAChT	rabbit	Synaptic system	139 103	AB_887864
**Secondary AB**	**Excitation**	**Maker**	**Cat #**	**RRID**
Goat anti-Guinea Pig IgG	488 nm	Invitrogen	A-11073	AB_2534117
Goat anti-Guinea Pig IgG	594 nm	Invitrogen	A-11076	AB_2534120
Goat anti-Mouse IgG	488 nm	Invitrogen	A-11029	AB_2534088
Goat anti-Mouse IgG	594 nm	Abcam	ab96881	AB_10680252
Goat Anti-Rabbit IgG	488 nm	Abcam	ab150081	AB_2734747
Goat Anti-Rabbit IgG	594 nm	Abcam	ab150084	AB_2734147
